# Overview of Recent Liquid Chromatography Mass Spectrometry-Based Methods for Natural Toxins Detection in Food Products

**DOI:** 10.3390/toxins14050328

**Published:** 2022-05-04

**Authors:** Annalisa De Girolamo, Vincenzo Lippolis, Michelangelo Pascale

**Affiliations:** 1 Institute of Sciences of Food Production (ISPA), National Research Council of Italy (CNR), 70126 Bari, Italy; annalisa.degirolamo@ispa.cnr.it; 2 Institute of Food Sciences (ISA), National Research Council (CNR), 83100 Roma, Italy; michelangelo.pascale@cnr.it

**Keywords:** natural toxins, food, analytical methods, liquid chromatography-mass spectrometry, simultaneous detection

## Abstract

Natural toxins include a wide range of toxic metabolites also occurring in food and products, thus representing a risk for consumer health. In the last few decades, several robust and sensitive analytical methods able to determine their occurrence in food have been developed. Liquid chromatography mass spectrometry is the most powerful tool for the simultaneous detection of these toxins due to its advantages in terms of sensitivity and selectivity. A comprehensive review on the most relevant papers on methods based on liquid chromatography mass spectrometry for the analysis of mycotoxins, alkaloids, marine toxins, glycoalkaloids, cyanogenic glycosides and furocoumarins in food is reported herein. Specifically, a literature search from 2011 to 2021 was carried out, selecting a total of 96 papers. Different approaches to sample preparation, chromatographic separation and detection mode are discussed. Particular attention is given to the analytical performance characteristics obtained in the validation process and the relevant application to real samples.

## 1. Introduction

**Figure 1:**
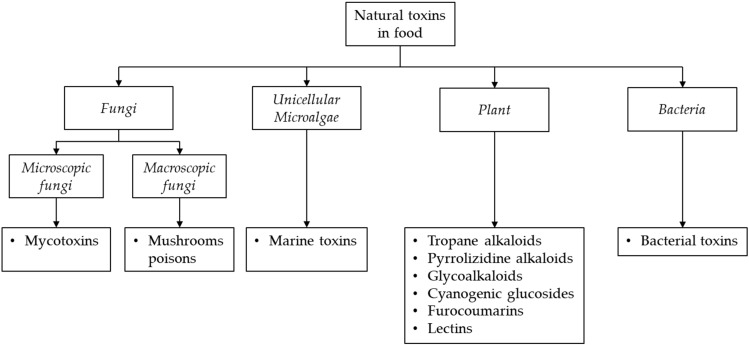
Classification of natural toxins occurring in food products based on their producing organisms.

Natural toxins include a wide range of toxic metabolites which are synthesized by various organisms such as animals, certain plant species or by microorganisms. They may endogenously occur when produced by organisms commonly present in food (resulting from the metabolism of a genus, species or strain) or exogenously occur when produced during the metabolism of living organisms [1,2,3]. In the last case, toxins occur in food as contaminants not intentionally added [4]. Some of them are present only in fresh crops and can be substantially removed by using appropriate processing, while others are unaffected by common food processing practices such as baking, cooking, and frying [5]. These toxins have different chemical structures, biological functions, occurrence, mode of action and toxicity and can cause a wide range of adverse health effects, including allergic or gastrointestinal reactions, and even death in the case of acute exposure. In the case of chronic exposure, immunosuppressive, reproductive, systemic or cancerogenic effects may occur [1,2,6]. Since the foods and consumed amounts included in daily diets are highly variable, consumers are constantly exposed to a large mixture of these natural toxins and at different levels, thus representing a great public concern. The exposure to the chemical mixtures may result in combined effects (i.e., additive, synergistic or antagonistic) depending on the dose of exposure. However, risk assessment of exposure of humans to complex mixtures of food natural toxins is still a great challenge [2]. When the toxins cannot be reduced or removed, intake should be limited. Several terminologies have been proposed for the classification of these natural toxins. According to the World Health Organization (WHO) and Food and Agriculture Organization (FAO) classification [1], the most common natural toxins posing a risk to human health include mycotoxins, produced by microscopic fungi (molds), mushrooms poisons, produced by macroscopic fungi, phytotoxins (including alkaloids, furocoumarins, lectins and cyanogenic glycosides), produced by various plant species, marine toxins, produced by unicellular microalgae, and toxins, produced by bacteria (Figure 1).

The reduction of risks related to the presence of natural toxins in food plays an essential role in protecting consumers. Indeed, WHO, together with the European Food Safety Authority (EFSA), FAO and Codex Alimentarius Commission, have established maximum residue limits (MRLs) or recommendations for many of these natural toxins to control their occurrence in food [7,8,9,10,11,12,13,14]. To ensure a correct risk assessment evaluation and compliance with the current legislation, it is important to develop sensitive, selective and robust analytical methods to determine the occurrence of these natural toxins in foodstuffs. However, the main challenge for the analysis of these compounds in food samples is related to their different physico-chemical properties, the inherent complexity of food matrices as well as the purpose of the analysis. These characteristics can affect the extraction efficiency of targeted toxins and then the accuracy and sensitivity of the method [4].

Different sample preparation strategies, including extraction, purification (clean-up) and preconcentration procedures have been proposed in the literature to eliminate possible interferences and enrich the sample. Solid phase extraction (SPE) and liquid–liquid extraction (LLE) have been more frequently applied for clean-up procedures. Among SPE, the most currently used extractive phases are those based on C18, polymeric and antibodies anchored onto a support material (immunoaffinity column, IAC) sorbent. Alternatively, the simple dilution of the sample (dilute and shoot) or protein precipitation have been used in the case of acceptable sensitivity of the method [4].

For the separation and final determination of natural toxins, methods based on high performance liquid chromatography (HPLC) coupled to fluorescence or ultraviolet (UV) detection have been widely used for the analysis of single or small groups of structural related toxins in food products [6]. HPLC-based methods have been evolving to more fast, efficient and environmentally friendly separations often involving ultra-high-performance liquid chromatography (UHPLC), multidimensional LC, capillary- and nano-LC systems providing an increased analysis throughput and performances.

The use of LC-based methods, which can lead to the coelution of some analytes, without providing any structural information, and the need to detect a great multiplicity of analytes in a single run, have shifted this field towards the use of LC in combination with mass spectrometry (LC-MS). Nowadays, LC-MS is the most powerful technique for the simultaneous detection of multiple regulated, unregulated, and emerging toxins in one single run due to its excellent sensitivity even at low concentration levels, selectivity, and its ability to resolve co-eluting compounds based on their molecular masses [15]. Most of the LC-MS methods employ different MS analyzers, such as triple-quadrupole (QqQ), ion trap (IT), time-of-flight (TOF) or orbitrap technology, depending on the kind of analysis, i.e., targeted or untargeted, and the required information. The use of UHPLC, multidimensional LC, capillary/nano-LC or even online sample preparation approaches have been widely applied to LC-MS analysis for natural toxins analysis [6,16].

The chromatographic separation of toxins is commonly carried out through reversed-phase columns, even though polar and ionizable analytes can better be retained/separated by other elution modes, such as hydrophilic interactions chromatography (HILIC). The LC-MS methods for the quantitative determination of natural toxins are commonly based on the use of triple-quadrupole analyzer, tandem mass spectrometry, with multiple reaction monitoring (MRM) mode, allowing to simultaneously analyze several compounds with high sensitivity, selectivity and accuracy [17]. To fulfil the EU confirmation criteria for LC-MS/MS-based methodologies, two MRM transitions have to be monitored for each analyte (parent and two-fragment ions) [17]. However, the use of triple quadrupole instruments is limited to a pre-defined number of compounds with known identity monitored within the analysis, and can be time-consuming due to the need for single standards analysis to optimize instrumental conditions. These limitations make LC-MS/MS methods based on the MRM approach not suitable to analyze unknown compounds. Nowadays, thanks to technological advances and more affordable prices, the use of high-resolution mass spectrometry (HRMS), mainly based on orbitrap MS and TOF MS systems, is growing, providing enhanced selectivity and allowing to screen and quantify many metabolites also including parent and unknown compounds in food and feed [16]. Moreover, the use of modern hybrid instruments combining two different types of analyzers, such as quadrupole linear ion trap (Q-LIT), quadrupole-TOF (Q-TOF), and quadrupole orbital ion trap (Q-Orbitrap) offers advantages in terms of selectivity and sensitivity.

The co-occurrence of natural toxins in combination with other chemical contaminants such as pesticides, growth regulators and veterinary drugs and bioactive compounds (i.e., lignans, flavonoids and phenolic compounds), in a broad range of food matrices has driven an increasing interest for analytical methods addressing the simultaneous determination of multiple analyte classes. The simultaneous determination of multiclass analytes in complex food matrices is commonly based on LC-MS analysis, either using triple quadrupole or high-resolution approaches, thanks to their advantages in terms of selectivity and sensitivity [18].

**Figure 2:**
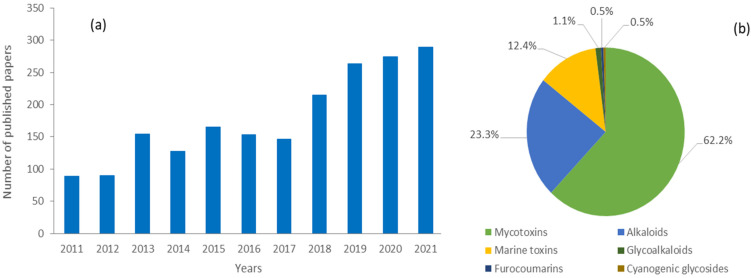
(**a**) Trend of published papers for the analysis of natural toxins (i.e., classes of mycotoxins, alkaloids, marine toxins, glycoalkaloids, cyanogenic glycosides and furocoumarins) in food by LC-MS methods from 2011 to 2021. (**b**) Fraction (%) of published papers for the selected classes.

This review focuses on the scientific progress in the application of LC-MS-based methods to the analysis of mycotoxins, alkaloids, marine toxins, glycoalkaloids, cyanogenic glycosides and furocoumarins in food, considering the literature published from 2011 to 2021. The six selected classes were those detected using a similar analytical approach and having a low or medium molecular weight. Proteins and (oligo)peptides (i.e., mushroom poisoning, lectins and bacterial toxins) were not included in this review. The literature search employed the Scopus online database (www.scopus.com, accessed on 31 January 2022) and keywords used for the search were “liquid chromatography mass spectrometry” and “food”; then, the keywords “mycotoxins“, “alkaloids”, “marine toxins”, “glycoalkaloids”, “cyanogenic glycosides” and “furocoumarins” were used to search the relevant literature for these particular groups of natural toxins in the selected years to identify all publications using LC-MS based methods. In this way, we found numerous articles, book chapters, and seminar proceedings. A total of 1966 papers were published in the selected period with a positive trend over the years and with mycotoxins representing the most abundant class (*n* = 1122), followed by alkaloids (*n* = 459), marine toxins (*n* = 244), and to a lesser extent glycoalkaloids (*n* = 22), furocoumarins (*n* = 10) and cyanogenic glycosides (*n* = 9) (Figure 2).

For all the classes, and in particular for the most abundant ones, a further refinement of selected papers was applied in order to select only those describing the development and validation of an LC-MS method (together with performance characteristics values). The novelty of the used technology, the application to new food matrices and the possibility to simultaneously analyze multiple toxins also including emerging ones were taken into account for the selection. In the case of mycotoxins, due to the high number of publications in the period 2011–2021, the literature search was refined to a more restricted period (i.e., from 2016 to 2021). A total of 96 papers were considered for this review by subdividing them in six specific sections, and in a further one for application to multiclass analysis.

## 2. Mycotoxins

Mycotoxins are a group of natural compounds produced under a wide range of climatic conditions by filamentous fungi mainly belonging to *Aspergillus*, *Penicillium*, *Fusarium*, *Claviceps* and *Alternaria* species. These toxins can contaminate various agricultural commodities (cereals, dried fruits, nuts, spices and coffee being the most frequently contaminated ones) either before harvest or under postharvest conditions, thus posing a risk to human and animal health due to their toxic effects. In addition, significant losses of yields and quality of the infested commodity are observed. Among the over 300 mycotoxins that have been identified, those of major concern worldwide causing food-borne illnesses are aflatoxins, ochratoxin A, fumonisins, zearalenone, patulin, citrinin, type B trichothecenes, mainly deoxynivalenol and nivalenol, and type A trichothecenes, mainly T-2 and HT-2 toxins. Their toxic effects range from nephrotoxicity, cytotoxicity, nervous-system disturbances, gastrointestinal diseases, to immunotoxicity, mutagenicity, teratogenicity and carcinogenicity [19,20]. In particular, aflatoxins have been classified by the International Agency for Research on Cancer (IARC) as carcinogenic to humans (Group 1) [20]. As a legislative consequence, maximum permitted or indicative levels have been established worldwide or are under discussion [7,9,21,22,23,24,25,26].

After the infection of crop plants, mycotoxins are modified by plant enzymes and are often conjugated to more polar substances. These substances are usually not detected during routine analysis, are unregulated, and are called “bound” or alternatively “hidden”, “conjugated” or “masked” and more recently “modified” mycotoxins. Some metabolites are more toxic than the parent compound, while others are less toxic. Furthermore, depending on the type of linkage with matrix component, a part of bound mycotoxins could become bioavailable again in the digestive tract of humans and animals, thus contributing to the toxicity of parent compound [27]. Other unregulated mycotoxins, that are usually not detected during routine analysis, but with some relevance from a safety and economic point of view, have also been reported in raw cereals and derived products. These compounds are known as “emerging mycotoxins”. The most prominent are beauvericin, enniatins, fusaproliferin, sterigmatocystin, citrinin, ergot alkaloids, moniliformin and *Alternaria* toxins [28,29,30,31,32]. Although not all the emerging mycotoxins are of concern to human health, the European Food Standard Authority (EFSA) has recently highlighted the need for long-term studies to assess potential chronic toxic effects. Furthermore, a continuous monitoring, collection of data, expertise and tools to set food safety standards is needed to understand and manage the potential risks of emerging mycotoxins in raw materials, feeds and foods [33].

Several chromatographic methods, mainly based on HPLC coupled with UV/diode array and fluorescence detection, have been developed and extensively reviewed for the determination of single mycotoxin or structurally related mycotoxins in food and feed. Gas chromatography (GC) and gas chromatography coupled to mass spectrometry (GC-MS) are also available for the specific detection of trichothecenes [34,35,36]. 

The evidence of co-occurring mycotoxins in various matrices has led to the development of new multi-mycotoxin methods for their simultaneous detection in the same matrix. The absence of structural information, as well as the coelution of structurally related mycotoxins, have shifted this field towards more sophisticated detection techniques based on the use of MS detectors. As a consequence, MS coupled with HPLC and ultra-HPLC systems has turned into one of the most powerful tools for multi-mycotoxin analysis at very low concentrations in complex matrices [36,37]. Furthermore, LC tandem mass spectrometry (LC-MS/MS), multi-stage mass spectrometry (MS^n^) and high-resolution MS (HRMS) can provide structural and accurate mass information not only for the determination of well-known mycotoxins with remarkable sensitivity and specificity, but also for the analysis of emerging and modified mycotoxins [18,36,38]. A recent review providing insight into LC-MS based methods for the simultaneous determination of mycotoxins indicated that in the period 2012–2016, approximately 80% of all published LC-MS methods on mycotoxins were based on LC-MS/MS, while more than 10% were LC-HRMS methods and the remaining 10% were LC-HRMS/MS [37]. Specifically, HRMS are becoming a relevant trend because they offer the possibility to perform target, post target, and non-target analysis in a single run.

The complexity of the matrices, mainly including those of animal origin, has led to the development and validation of specific analytical protocols allowing for quantitative extraction and detection of targeted mycotoxins. The availability of standardized methods of analysis is of paramount importance to guarantee a uniform application of the EU legislation and contribute to maintaining a high level of food and feed safety. Despite the huge number of published multimycotoxin LC-MS methods, their implementation in control laboratories has been limited in the past years due to their performance characteristics not fulfilling the acceptability criteria for mycotoxins established at the EU level [34]. In 2013, the European Commission (EC) invited the European Committee for Standardization (CEN) to develop and standardize 11 methods for mycotoxins determination in food within the EC standardization mandate M/520 [39]. Among them, six methods based on the use of LC-MS/MS have been recently adopted as CEN-EN-ISO standard methods (https://standards.cencenelec.eu/ accessed on 5 April 2022). 

### LC-MS Methods for Mycotoxin Determination in Food

Table S1 shows examples of published LC-MS methods for multi-mycotoxin determination in foods worldwide during 2016–2021. A total of 28 papers were selected covering from 2 to 33 well-known detected mycotoxins in a wide range of matrices, including cereals and derived products, as well as beer, edible oils, milk, medical herbs and dried fish. The most used sample preparation approaches included SPE column clean-up or alternatively the Quick, Easy, Cheap, Effective, Rugged and Safe (QuEChERS) approach. The most frequently used MS technology was the QqQ in MRM mode. Some examples of Q-LIT, Q-TOF and Q-Orbitrap were also reported as alternative MS approaches for the analysis of these natural toxins. 

A multi-mycotoxin analysis method based on UPLC-ESI-MS/MS for the determination of 26 mycotoxins, including both well-known and emerging mycotoxins, in durum wheat was proposed by Juan et al. [40]. Accuracy and precision values were in compliance with the EU Regulation 519/2014 [41], while sensitivity values (i.e., limits of quantification, LOQs) were lower than the MRL established by the EC Regulation 1881/2006 [7] for durum wheat. The method was applied to evaluate the (co)-occurrence of targeted mycotoxins in 74 durum wheat samples harvested in central Italy.

The simultaneous detection of five types of *Fusarium* mycotoxins in processed grains was described by Kai et al. [42] using an LC-MS/MS method. The multi-functional SPE procedure was used for the clean-up of sample extracts obtained using an acetonitrile–water mixture as extraction solvent. The in-house validated method applied to 55 food samples, including wheat and corn, showed the co-occurrence of targeted mycotoxins.

Another paper, published by Sharmili et al. [43], described a simple, fast and reliable method applying a QueEChERS extraction procedure followed by dispersive SPE (d-SPE) clean-up and LC-MS/MS for the simultaneous determination of aflatoxins, ochratoxin A, deoxynivalenol and zearalenone in vegetable oil. The developed method was validated *in house* according to the criteria of National Public Health Laboratory’s standard operating procedure No: A03-005 for Method Validation in Chemical Analysis and then applied to the analysis of 25 commercial vegetable oil samples from Malaysia. 

A simple, rapid and accurate method based on QueEChERS followed by SPE C18 columns and detection with UPLC–MS/MS was optimized and validated for the simultaneous determination of 25 mycotoxins in cereals [44]. The optimization procedure focused on the selection of clean-up sorbents, extraction solvent, chromatographic conditions and on the matrix effect removal. Performance values were evaluated in terms of precision, accuracy and sensitivity and the method was applied to the analysis of 65 samples of cereals, including wheat, corn and rice, from different areas of China. Wang et al. [45] described the development and validation of a fast method based on a d-SPE clean-up, using C18 sorbent and UPLC-Q-TOFMS, for the simultaneous determination of nine mycotoxins in corn. Limits of detection and quantification were below the EU MRL [7]. The developed method was applied to the analysis of 130 Chinese corn samples, and fumonisins were the main class of occurring mycotoxins. 

Another method was developed for the simultaneous determination of 21 mycotoxins in white peony root, Radix Paeoniae Alba (RPA), by using a QuEChERS-based approach followed by d-SPE (C18 sorbent) and detection by UHPLC Q-LIT MS [46]. In particular, a MRM information-dependent acquisition-enhanced product ion scan mode was used, and 13 mycotoxins were detected in positive mode while the other mycotoxins were analyzed in negative mode. The developed method was applied to the analysis of 20 RPA samples, showing about 65% of contamination incidence.

Zwickel et al. [47] reported an LC-MS/MS method for the simultaneous quantification of 12 *Alternaria* toxins in wine, vegetable juices and fruit juices and presented for the first time analytical data for altenuic acid, altenuisol and iso-altenuene. Furthermore, an improvement of the chromatographic performance was achieved for tenuazonic acid compared to previously reported multi-analyte methods for *Alternaria* targeted toxins in foodstuffs. Validation results were compliant with the requirements reported in the CEN technical report on the performance criteria for single laboratory validated methods of analysis for the determination of mycotoxins [48]. The validated method was applied to the analysis of 103 commercial wine and juice samples collected from Germany, with red wines and grape juices being the most frequently contaminated matrices with multiple *Alternaria* toxins.

Al-Taher et al. [49] developed an LC-MS/MS method for the detection of 11 mycotoxins in infant cereals, including rice, barley, oats and mixed grains. Specifically, the method was based on the use of a single-step extraction and a stable isotope dilution assay. The use of UHPLC allowed a good separation of mycotoxins in less than 10 min, which is crucial for high throughput screenings. The method was validated *in house* in terms of sensitivity, linearity and accuracy, and successfully applied to the analysis of targeted mycotoxins in 64 infant cereals samples from the U.S. market. 

In another study, a fast, easy and cheap method was developed for the determination of eight mycotoxins in cereal-derived products (wheat flours, dry pasta, baked foods, corn meals and breakfast cereals) based on the use of QuEChERS extraction and LC-MS/MS analysis [50]. Performances of the validated method on wheat flour samples fulfilled the criteria set by the EU legislation [41]. The method was applied to the analysis of 21 cereal-derived products from the Italian market, showing the co-occurrence of some targeted mycotoxins. 

A method based on LC-MS/MS was developed and validated for the analysis of 11 mycotoxins in edible oils [51]. A simple solvent extraction approach followed by a defatting process using n-hexane was employed for a fast and high-throughput analysis. Performance of the validated method fulfilled acceptance criteria established at the EU level [52]. Although the method was applied to the analysis of only nine Korean edible oils, authors stated that additional studies involving larger sample sizes were needed to assess the safety of this category of samples.

Another example of multiclass mycotoxin analysis was proposed also for cow milk samples by Flores-Flores and Gonzalez-Penas [53]. Specifically, an LC–MS/MS method was validated for the simultaneous analysis of 15 mycotoxins in cow milk, including those that have not been frequently studied in this matrix, such as fumonisins, sterigmatocystin or ochratoxin B. The sample preparation consisted of an LLE using acidified acetonitrile, and a clean-up of the extract with sodium acetate. The method was applied to the analysis of 10 Spanish cow milk samples. No mycotoxins were found, and the authors concluded that more samples should be analyzed to evaluate their presence in this matrix.

Kim et al. [54] developed an analytical method for the simultaneous determination of 13 mycotoxins in cereal grains by LC-MS/MS after multi-mycotoxin IAC clean-up. The one-step elution provided high recovery values for all tested mycotoxins. The validated method was used in a large survey using more than 500 samples of brown rice, maize, millet, sorghum, and mixed cereals collected from local markets in South Korea. *Fusarium* mycotoxins were the most frequently and co-occurring detected mycotoxins in the investigated samples.

An innovative approach to multi-class mycotoxin control was proposed by Arroyo-Manzanares et al. [55] for the analysis of 21 mycotoxins produced by *Aspergillus*, *Fusarium*, and *Penicillium* fungi and 12 ergot alkaloids produced by Claviceps purpurea in wheat and maize. The simple extraction procedure based on the “salting-out” LLE, minimizing the epimerization of ergot alkaloids, in combination with UPLC-MS/MS analysis, were the key factors for the rapid, robust and sensitive detection of these targeted compounds. The method was successfully applied to study the co-occurrence of targeted mycotoxins in 28 wheat and corn samples collected from six European countries.

The feasibility of using an ultra-high-performance supercritical fluid chromatography (UHPSFC) and MS/MS as separation tool for 15 *Fusarium* mycotoxins analysis, including their modified forms, in beer has been described [56]. The sample preparation included an LLE and SPE C18 columns clean-up. Although they observed a limited applicability of UHPSFC to the routine multimycotoxin analysis, the authors suggested this approach as a separation technology for isomers to be used in the mycotoxin–biomarker field.

In another paper, Du et al. [57] described the use of a rapid microwave-assisted dispersive-micro-SPE (MA-d-µ-SPE) and subsequent analysis by UHPLC-Q-TOF-MS for the determination of six mycotoxins in peach seed, milk powder, corn flour and beer. Several parameters of the d-µ-SPE were compared and optimized with nano zirconia selected as the optimal dispersant. The validated method provided limit of detection (LOD) values lower than the EU MRLs [7].

Li et al. [58] described the use of UPLC-MS/MS for the determination of 16 mycotoxins in maize. An *ad hoc* SPE clean-up was optimized by comparing four different approaches with Mycospin 400 column enabling acceptable recoveries for all mycotoxins including ochratoxin A and sterigmatocystin, which showed lower recoveries in the other tested protocols. The application of the developed method on 80 maize samples collected from Shandong Province in China showed that more than 70% of samples were contaminated with at least one of targeted mycotoxins. 

Another application of UPLC-MS/MS was described for determination of citrinin and ochratoxin A in a variety of food and feed matrices [59]. A QuEChERS-based extraction and 5-fold concentration were proposed as a sample preparation protocol. The developed method was validated using the criteria described by Commission Regulation No. 401/2006/EC [52] and Commission Decision No. 2002/657/EC [17] as guidance. The validated LC–MS/MS method was applied to evaluate the occurrence of target toxins in 90 Belgian foodstuffs.

A comparison of different approaches commonly used for the LC-MS/MS analysis of 12 mycotoxins in cereal foods was carried out by Solfrizzo et al. [60]. In particular, the study compared 12 analytical methods with different extraction solvents, purification strategies (i.e., SPE, QuEChERS, and IAC), and calibration approaches (i.e., external or matrix-matched). The method providing the best results, based on water/methanol extraction followed by two consecutive IAC clean-up and external calibrations, was further validated *in house* on corn, rice and feed according to the EC Regulation 401/2006 [52]. Performance in terms of accuracy and precision made this method suitable for regulatory purposes even when using lower-performing LC-MS/MS apparatus. 

A modified QuEChERS method combined with nano flow LC-HRMS was proposed by Alcantara-Duran et al. [61] for the determination of 17 mycotoxins in peanut, almond and pistachio. After comparing two purification procedures, the d-SPE using Enhanced Matrix Removal lipid reduced the matrix effect and improved recoveries for all mycotoxins. The method, validated according to SANTE guidelines [62], was applied to the analysis of investigated mycotoxins in 15 samples collected in Spain.

Dong et al. [63] described the development of a single-step SPE for the analysis of seven mycotoxins in fruit and vegetables by UHPLC-MS/MS using the dynamic MRM approach. The validated method was applied to the analysis of cherry tomato, leafy vegetables, strawberry and tomato samples. Although none of the targeted mycotoxins were detected in fresh samples, the concentration of alternariol, alternariol methyl ether and tenuazonic acid increased after storage experiments.

Another comparative study was carried out by Scarpino et al. [64] that developed and compared two clean-up methods for the determination of 17 *Fusarium* and *Aspergillus* mycotoxins, including emerging and masked mycotoxins. Specifically, the dilute-and-shoot and SPE clean-up approaches were compared in combination with LC-MS/MS analysis. Results, in terms of precision, accuracy and reliability, indicated that both methods, thanks to the reduction of time and cost of the analysis, were promising for high throughput routine multimycotoxin analysis. It was the first time that Oasis^®^ PRiME HLB columns were applied for the clean-up of the targeted mycotoxins considered in the study.

Recently, Woo et al. [65] described the optimization and validation of an LC-MS/MS method after comparing three different sample preparation procedures (i.e., SPE, QuEChERS, and IAC) for the simultaneous analysis of 20 mycotoxins in doenjang, a Korean fermented soybean paste. The method based on IAC clean-up provided the best results in terms of linearity, precision, recovery, matrix effect, and measurement uncertainty. The validated method was subsequently applied to the analysis of targeted mycotoxins in 60 samples of commercial and homemade doenjang. More than 80% of doenjang samples were contaminated by at least one toxin, with the highest levels and number of co-occurring mycotoxins in homemade products compared to in commercial ones. 

An LC-MS/MS method for the simultaneous determination of seven major trichothecenes in wheat, wheat flour and wheat crackers was recently validated by a collaborative study involving 15 participant laboratories [66]. This study was carried out within the M/520 standardization mandate of the European Commission and performed according to the AOAC/IUPAC International Harmonized Protocol [67]. The method was based on acetonitrile–water extraction followed by SPE purification for the sample preparation and on the use of isotopically labelled mycotoxins as internal standards for the LC-MS/MS analysis. The validated method has been adopted as a CEN standard method [68].

The necessity to monitor mycotoxins also in dried seafood products has recently led to the development of a sensitive, selective and accurate LC-MS/MS for the quantification of aflatoxin B1, T-2 toxin, ochratoxin A and deoxynivalenol in these specific matrices [69]. After comparing different sample preparation approaches, the optimized method was based on an ultrasound-assisted acetonitrile/water extraction followed by a defatting clean-up with n-hexane. The analysis of 40 dried fish, dried shrimps and dried mussels samples highlighted for the first time a high occurrence of these mycotoxins in samples collected in the Zhanjiang region. 

Gbashi et al. [70] proposed for the first time the use of pressurized hot-water extraction (PHWE) methodology for the LC-MS/MS analysis of 15 mycotoxins in maize. Moreover, a chemometric approach, based on central composite design, was used for the optimization of the extraction conditions. The validated method was tested on 25 household maize meal samples from South Africa, with fumonisin B1 being the contaminant with the highest occurring frequency and contents. Although the method PHWE is a promising, suitable, cost-effective and greener alternative to traditional methods, further studies are needed to evaluate the cost-benefit of using this approach.

Very recently a rapid and sensitive QuEChERS-UPLC-QTOF method, based on matrix-matched calibration, was developed and validated for the determination of 17 mycotoxins, including emerging ones, in malted barley and beer [71]. Validation parameters of the method were evaluated according to the International Conference of Harmonisation (ICH) [72] while the uncertainty associated was estimated according to EURACHEM [73]. For the EU-regulated mycotoxins, performance parameters fulfilled the EU acceptance criteria [41]. 

A dispersive liquid–liquid microextraction in combination with LC-MS/MS analysis was described for the simultaneous determination of 12 mycotoxins in rice bran [74]. The method was optimized by using the multivariate statistical techniques based on response surface methodology in combination with a Box–Behnken design. The optimized method was validated according to the EU Regulation 2002/657 EC [17] showing satisfactory validation characteristics. The method was applied to the analysis of 24 rice bran samples, with a rate of 42% of positive samples.

Finally, a recent study has proposed a multi-mycotoxins immunoaffinity column (multi-IAC) and LC-MS/MS method to evaluate 10 mycotoxins in traditional Chinese medicinal materials (TCMMs) [75]. The method was validated for linearity, precision, recovery, analytical limits, and matrix effect. Furthermore, the method was successfully applied to systematically investigate the co-occurrence and contamination levels of multi-mycotoxins in 30 TCMs and functional foods that were all positive for aflatoxin B1. Despite a limited number of samples, this study showed a great multi-mycotoxins contamination rate (more than five different mycotoxins) in TCMMs.

## 3. Alkaloids

Alkaloids are a group of amino-acid-derived and nitrogen-bearing molecules produced by several plant species that serve as a natural defense against aggression from other organisms such as insects or herbivores [76]. Alkaloids can be distinguished into pyrrolizidine alkaloids (PAs) and tropane alkaloids (TAs) [77]. The group of PAs comprises more than 600 pyrrolizidine alkaloids and N-oxide derivatives (which act as distinct compounds with contrasting physical properties) that are mainly found in three plant families, namely *Boraginaceae*, *Asteraceae,* and Legumionsae (*Fabaceae)* and about half of them affect wildlife, livestock and humans. PAs have also been evaluated as undesirable substances in food and feed by the EFSA [78]. The toxic effects of PAs are principally on the liver. Indeed, although the acute disease is associated with high mortality, a subacute or a low long-term exposure to PAs leads to cirrhosis of the liver. Several PAs and PA-containing plant materials were evaluated by IARC and lasiocarpine, riddelliine, and mono-crotaline were classified by the IARC in Group 2B (possibly carcinogenic to humans), while isatidine, retrorsine, and senkirkine were included in Group 3 (not classifiable) [20,77,79]. Based on the present knowledge, it was concluded that 1,2-unsaturated PAs may act as genotoxic carcinogens in humans. Food contaminated with PA plant material, such as honey, grains, milk, tea, infusions, herbal remedies and dietary supplements are the most common sources of PA poisoning. To protect public health, the EC Regulation n. 2020/2040 of 11 December 2020 defines maximum levels for 21 PAs in certain foodstuffs, and it will be applied from 1 July 2022 [8]. In the case of TAs (such as atropine and scopolamine) and their corresponding N-oxides derivatives, the main producing plant families are *Brassicaceae, Solanaceae* and *Erythroxylaceae*. The (-)-enantiomers hyoscyamine and scopolamine are the most studied TAs which, in contrast to the (+)-enantiomers, are naturally formed. The racemic mixture of (-)-hyoscyamine and (+)-hyoscyamine is called atropine [80]. Although more than 200 different TAs have been identified in various plants, toxicity and occurrence data in food and feed are limited. Several TAs (e.g., scopolamine) are hallucinogenic and some are powerful anticholinergic drugs (e.g., atropine, hyoscyamine, scopolamine). Although human intoxications by TAs result mainly from abuse of TA-containing plants such as *Datura stramonium*, EFSA concluded that there is no information available on the carry-over of TAs from feed into animal products, such as milk or tissues from exposed animals, except for traces of alkaloids that have been found in eggs [77]. A study carried out by Adamse and van Egmond [81] concluded that human foods potentially containing TAs would be tea and herbal preparations and that there are products, like buckwheat, that should primarily be monitored to prevent accidental exposure of humans to TAs. With the EU Regulation 2016/239, maximum limits for atropine and scopolamine have been defined in certain cereal-based foods for infants and young children and new maximum limits are foreseen to be extended also to other cereals, pseudocereals and derived products, and herbal infusions [82]. Furthermore, in 2013, EFSA derived an acute reference dose (ARfD) for the sum of atropine and scopolamine and concluded that for toddlers, the group ARfD for the tropane alkaloids atropine, (-)-hyoscyamine, and (-)-scopolamine can be exceeded through the consumption of cereal products [83]. In the same year, the Bundesinstitut für Risikobewertung (BfR) received a mandate from the Federal Ministry of Food and Agriculture to check whether the ARfD of 0.016 µg/kg body weight for TAs derived by the EFSA can be used as the basis for a risk assessment for food. The conclusion was that further efforts should be given to improve the database on the occurrence of TAs in different food categories and on their consumption [84].

Analytical methods for pyrrolizidine alkaloids (PAs) and N-oxide derivatives are typically based on GC-MS or LC-MS determinations. GC-MS methods commonly require the reduction of N-oxide derivatives and a subsequent derivatization step prior to chromatographic analysis. Other methods quantify total PAs by reducing PAs to necine pyrroles without any structural discrimination. On the other hand, the use of LC-MS analysis permits the quantification of both PAs and relative N-oxides without reduction steps [85,86]. Classical analysis of tropane alkaloids (TAs) has been mainly focused on the determination of atropine and scopolamine by capillary electrophoresis, even though this technique shows low sensitivity. More common methods are chromatographic ones, i.e., LC or GC, coupled with different detectors. Although GC methods offer a good chromatographic separation, the derivatization of TAs with trimethylsilyl is needed due to thermolysis. LC analysis, in combination with UV/DAD detectors, overcomes this problem. As coupling LC with MS detectors offers a better sensitivity and selectivity and a good chromatographic separation (except for enantiomers), this technique is being commonly employed to analyze TAs in food matrices [87,88]. For these reasons, LC-MS/MS are currently the predominant approaches for analysis of PAs or TAs, as well as for their simultaneous determination. However, LC-MS/MS technologies, usually acting in MRM acquisition mode, operate at a relatively low resolution and cannot discriminate structurally similar or isobaric compounds that could produce the same mass fragments and thus, causing ambiguity in identification. Furthermore, the notable limit of LC-MS/MS is related to the impossibility to carry out retrospective data evaluation that is useful for the screening of unidentified compounds. On the contrary, the use of HRMS technologies, and in particular those using hybrid approaches like Q-Orbitrap or Q-TOF, enables ultra-high resolving power values, and are very promising in the analysis of this class of natural toxins [89]. Moreover, multistage fragmentation (MS^n^) or differential ion mobility mass spectrometry can be considered very helpful in discriminating isobaric compounds [90]. In June 2015, the European Commission issued the Recommendation (EU) 2015/976 on the monitoring of the presence of TAs in food and on the method of analysis that should preferably be HPLC-MS/MS or, if not possible, GC-MS. Furthermore, recommended LOQ values for scopolamine and atropine in foods have been given [91].

### LC-MS Methods for Alkaloids Determination in Food

Table S2 shows examples of published LC-MS methods for the detection of PAs, TAs and relative N-oxides in foods worldwide during the last decade, 2011–2021. A total of 21 papers were selected covering from 2 to 54 alkaloids mainly in honey, herbal tea, animal-derived products, cereals and cereal-based products. Except for two papers analyzing both PAs and TAs, the others investigated only a single class of alkaloids. The majority of selected papers used SPE column clean-up for the sample preparation and the LC-MS/MS with MRM detection mode.

Jakabova et al. [92] described the development of a method for the determination of atropine and scopolamine in plant organs (i.e., stems, leaves, flowers, fruits and seeds) of different *Datura* species by LC-MS. The use of a simple sample preparation step (i.e., ultrasound assisted SLE with methanol/water mixture), together with the use of a new generation of core-shell particle packed LC column, made the method simple and fast. The method was validated in house and applied to determine the targeted toxins in dry-plant materials of four different *Datura* species, with higher contamination levels in samples collected in autumn.

A method allowing the detection of 11 pyrrolizidine alkaloids in honey by LC-ITMS was described by Griffin et al. [93]. An SPE clean-up through polymeric SCX sorbent offered good recoveries of toxins. Furthermore, the automated identification based on spectral library matches allowed the accurate data analysis and the quick and robust detection of targeted alkaloids in honey samples. Then, a screening of 50 retail honey samples, collected in Ireland, was carried out using the validated method. A total of 16% positive samples was observed. 

Mudge et al. [94] proposed a method based on SPE clean-up, with SCX sorbent, and LC-MS analysis for the quantification of five pyrrolizidine alkaloids in plant materials and honey samples. After optimization of extraction conditions (i.e., extraction solvent and extraction modes), the method was subjected to a single laboratory validation according to the AOAC International guidelines [95]. The acceptable performance parameters made this method suitable for the analysis of the targeted analytes in officinalis plant parts and in honey collected in North America.

An innovative, fast and specific method was described for the determination of anisodine, scopolamine, anisodamine and atropine by LC-MS/MS in *Przewalskia tangutica* Maxim. fruit extracts, a medicinal plant found in the Tibetan Plateau of China [96]. The authors described the preparation of a molecularly imprinted polymer (MIP) using anisodine as a template, and its application to the selective extraction and preconcentration of the targeted TAs.

Another application to honey was reported by Lorena et al. [97] for the determination of seven PAs (i.e., echimidine, heliotrine, lycopsamine, retrorsine, senecionine, seneciphylline) and their N-oxides in honey by SPE clean-up (with SCX sorbent) and LC-MS/MS detection. The use of a reduction step with zinc dust and sulphuric acid before the SPE allowed the complete reduction of N-oxides to their corresponding free bases. The method was validated according to the EU Directive 2002/657/EC [17] and then applied to the analysis of 60 Italian honey samples. A total of 17% of samples were positive to at least one of the monitored PAs. 

Valese et al. [98] developed a fast and simple LC-ESI-MS/MS for the determination of 7 PAs in honey. The simple water-dilution protocol before instrumental analysis made the method low-cost, green and high-throughput (11 min analysis time). The validation carried out in agreement with the EU Directive 2002/657/EC [17] provided satisfactory performance results. Its applicability was tested by the analysis of the targeted PAs in 92 Brazilian honey commercial samples, and more than 99% of them contained at least three PAs.

The determination of atropine and scopolamine in buckwheat and derived products, soy, wheat, millet and chia seeds by UHPLC-ESI-HRMS has been described [99]. The clean-up step consisted of a QuEChERS combined with a d-SPE with primary secondary amine (PSA) sorbent that was selected after comparing the effect of different sorbents. The validated method was applied to the analysis of samples of buckwheat, wheat, soy, buckwheat flour, buckwheat noodle, amaranth grain, chia seeds and peeled millet from Spain. Although no targeted TAs were found in the investigated samples, the use of HRMS allowed the identification of three scopolamine modified compounds (i.e., norscopine in amaranth, hydroscopolamine and dihydroxyscopolamine in chia seeds).

In the same year, Martinello et al. [89] described the development and validation of an analytical LC- HRMS method for the determination of two TAs and nine PAs in honey using a fast and simple QuEChERS and d-SPE (primary secondary amine and magnesium sulphate) protocol for sample preparation. The validation of the method was carried out according to the Commission Regulation (EC) N. 333/2007 [100], and relevant amendments, and the obtained LOQ values of scopolamine and atropine fulfilled the requirements of the EU Recommendation [91]. Then, it was applied to the analysis of 40 acacia and multifloral honey samples commercialized in Italy. Results showed that at least one PA or TA was detected in 70% of samples, with echimidine as the most abundant PA.

In another study, a UPLC-MS/MS analytical method was proposed for the determination of 15 PAs and 13 respective N-oxides in different groups of food [90]. The ultrasonic assisted SLE and the clean-up through the C18 SPE column allowed the application of the method to several matrices including cow milk, tea infusion, honey, cooked chicken, egg, cooked beef, barley flour and clove leaves. The use of MRM transitions, multistage fragmentation (MS^3^), and MRM with differential ion mobility spectrometry (DMS) were evaluated and compared in terms of selectivity and applicability. The DMS+MRM mode provided better results. The method using isotopically labeled PAs was validated according to the EURACHEM Guide [73] and showed good performance results.

Cirlini et al. [101] described the detection of atropine and scopolamine in organic buckwheat and derived products by UHPLC–MS/MS after a simple dilution of the extracted samples. Different extraction solvent mixtures were compared to minimize processing steps and reduce the time needed for sample preparation. The final optimized method, using an acidified methanol/water mixture, was successfully validated according to ICH [72], thus proving to be reliable and useful for screening traces of TAs in buckwheat and derived products. A preliminary survey, carried out on 26 commercial Italian samples of buckwheat organic products (i.e., flours, pasta, bakery products), showed the presence of TAs in three samples. 

The development and validation of an LC-MS method for the determination of 10 PAs in honey was described by Kowalczyk and Kwiatek [102]. After comparing different cation exchange SPE sorbents, the optimized method was based on the use of MCX columns (mixed-mode strong cation-exchange sorbent). The developed method was successfully validated according to SANTE/11945/2015 requirements [103] and applied to 50 Polish real honey samples, with 32% of samples positive for at least one PA.

Recently, an LC-HRMS method, based on SPE purification (with SCX sorbent), was described by Hungerford et al. [104] for screening of 30 PAs, also including their N-oxides, in honey. The use of low temperature (5 °C) in the UPLC chromatographic conditions was effective in resolving the key isomeric alkaloids, such as indicine and lycopsamine. The method was validated according to the National Association of Testing Authorities guidance document [105]. The large evaluation, carried out on 465 Australian honey samples, showed that the predominant PAs were lycopsamine, indicine and intermedine, and also identified Parsonsia vines as a previously unsuspected source of PA contamination in Australian honey.

The use of LC-HRMS was also described by Ji et al. [106] for the determination of 11 PAs in Gynura procumbens, an edible herb that has been approved as an ingredient for food and dietary supplements in China and that is commonly used also as a vegetable. Different SPE columns were compared for sample clean-up with Cleanert PCX (polymer based) columns providing the highest recoveries. After validation, the method was successfully applied to the analysis of PAs and its derived products in the aerial part of 12 *G. procumbens* and 7 commercial finished products. Results indicated for the first time that the occurrence of the targeted toxins in this plant material at variable levels might be depending on the geographical origin of the herb. 

Wang et al. [107] developed an LC-HRMS method that allowed simultaneous quantification of 12 PAs in honey. Specifically, two different analytical approaches were investigated and combined in the proposed method. Firstly, a method that allowed the simultaneous quantification of the targeted PAs in honey using LC-QTOF-MS with pure authentic standards was developed and validated according to the Nordic Committee on Food Analysis [108]. Then, a multi-target screening approach based on a semi-quantification strategy was used to evaluate the concentrations of other PAs without available standards (not included in the validation method) using a quantitative prediction model. The prediction model was subsequently validated by cross-validation (leave-one-out), obtaining a maximum concentration prediction error of 50.8%.

In another study, Zheng et al. [109] described a modified QuEChERS method for the determination of scopolamine, L-hyoscyamine and sparteine in animal-derived products by LC-MS/MS. Different protocols for the purification of the three TAs in the tested matrices were compared, and the QuEChERS d-SPE (C18 sorbent) mixture was selected as the most appropriate for the analysis of these analytes in animal matrices. The method was validated and applied to the analysis of 30 samples of porcine muscle, chicken eggs, and milk commercialized in the Republic of Korea. 

Basle et al. [110] describes a simple LC-MS/MS analytical procedure for the analysis of atropine and scopolamine in cereals and cereal-based products. The sample preparation was carried out using QuEChERS and a defatting protocol with n-hexane. The procedure was validated in maize grain and infant cereals according to the European SANTE/12682/2019 document [111] and performance parameters in terms of precision and recovery fulfilled its requirements. The method was further applied to 95 cereals and cereal-based products collected from Asian and African countries, and 29 of these samples were also analyzed for mycotoxins. 

A novel LC-MS/MS method was developed for the simultaneous determination of 21 TAs and 33 PAs together with their N-oxides in plant-based food matrices, such as sorghum, oregano, and mixed herbal tea using d-SPE clean-up [112]. All the targeted alkaloids were those considered of concern from EFSA evaluations [78,79]. For the sample preparation, the SLE with an acidified methanol/water extraction solvent and a d-SPE clean-up were used. The use of a reversed phase UHPLC-MS/MS enabled the accurate determination of 49 of 54 targeted alkaloids, while lycopsamine, echinatine, and N-oxides of indicine and intermedine, which could not be resolved on the commonly used RP column, were separated in a complementary HILIC system. The validated method on sorghum, oregano and herbal tea achieved performance characteristics that were in accordance with SANTE/12682/2019 method specifications [111]. 

Recently, the European Commission’s Joint Research Centre proposed an LC-MS/MS analytical method for the determination of atropine and scopolamine in cereal-based foods for infants and young children, tea and herbal infusions [113]. The method was previously validated *in house* in black tea, peppermint and fennel and then two proficiency tests (PTs), addressing the determination of targeted TAs, were performed. This was the first time in which a subset of PTs participants strictly followed the specified analytical protocol provided by the coordinator and these data were additionally exploited to derive interlaboratory performance characteristics indicating that the method was a good candidate for standardization.

Kaczynski and Lozowicka [114] described a novel and fast method based on LC–MS/MS for the simultaneous determination of 30 PAs and their corresponding N-oxides in herb samples. QuEChERS extraction technique combined with ultrasound-assisted d-SPE graphene provided appropriate conditions for the separation and quantification of targeted PAs in complex matrices such as herb materials. Full validation of the method was performed according to the SANTE/12682/2019 document [111] on peppermint, chamomile, nettle and linden and applied to test 50 real Polish herbs (chamomile, linden, nettle and peppermint) with chamomile and peppermint being the only matrices contaminated by PAs and relent N-oxides.

A simple and sensitive method using HILIC-MS/MS was developed for the determination of atropine and scopolamine in honey [115]. A sample preparation protocol was optimized based on salting-out assisted LLE. Moreover, the combination of HILIC and MS/MS techniques permitted an enhanced sensitivity for the two TAs. The in-house validated method was applied to 23 samples of honey and only two of them were found to contain residues of the TAs at concentrations above the LOQs.

Recently, an LC–MS/MS method coupled with 50% methanol extraction and MCX-SPE purification was developed for the determination of 28 PAs in two herbal medicines, *Tussilago farfara* and *Lithospermi erythrorhzion* [116]. The analytical method was validated according to the AOAC guidelines for validation of the botanical identification method [117]. The developed method was applied to determine the content of 28 PAs in 20 Chinese herbal medicine samples.

## 4. Marine Biotoxins

Harmful algal bloom (HAB) is a natural phenomenon caused by the overgrowth of marine phytoplankton under certain environmental conditions. Specifically, this phenomenon has been increasing due to rising ocean temperature and growing coastal eutrophication [118]. About 300 microalgal species are involved in the HAB events and among them more than 100 of these species produce persistent natural toxins, called marine biotoxins, that can cause significant food safety risks for humans when accumulated in shellfish. The main species producers of marine biotoxins dangerous for humans are those belonging to the genera *Alexandrium*, *Gymnodinium*, *Dinophysis*, and *Pseudo-nitzschia*. Marine toxins can accumulate in the tissue of filter feeding bivalves, including mussels, clams, scallops and oysters. On the basis of their poisoning symptoms, these toxins are classified as paralytic shellfish poisoning, amnesic shellfish poisoning, diarrhetic shellfish poisoning, neurotoxic shellfish poisoning, and ciguatera fish poisoning. Furthermore, marine biotoxins have different solubility and can be hydrophilic, including domoic acid, or lipophilic toxins. Major lipophilic toxins include okadaic acid, dinophysis-toxins, azaspiracids, pectenotoxins, yessotoxins and cyclic imines, which include spirolides, pinnatoxins, pteriatoxins and gymnodimines. 

For many years, mouse bioassays (MBA) have been used as reference methods to test for the presence of marine biotoxins in the food items, mainly for lipophilic and paralytic shellfish poisoning toxins. However, MBA tests show lack of specificity, poor sensitivity, false positives and are time-consuming, in addition to ethical concerns. Therefore, MBA and other biological methods have been used only for the detection of new or unknown toxins and not for routine analysis [6]. Several analytical methods have been proposed and used as alternative approaches to MBA, mainly those based on HPLC with UV/diode array or fluorescence detection, and more recently, on LC-MS/MS techniques [6]. In the last decade an increasing attention has been paid to the development of multitoxin LC-MS methods with specific attention to the optimization of sample preparation, sensitivity (up to ppb levels) and rapidity to permit screening of these toxins in shellfish and derived products. These methods have been used for quantitative determination of those marine biotoxins relevant for which regulatory limits have been set. Indeed, the EU Regulation stated that LC-MS/MS-based methods are the reference methods for the detection of lipophilic toxins in shellfish while MBA can be used as alternatives or supplementary to LC-MS/MS methods [119]. In general, LC-MS analysis of marine biotoxins is commonly performed on triple-quadrupole MS operating in MRM mode [120]. Due to the targeted nature of MRM, only known toxins can be detected, while new or modified biotoxins could remain undetected indefinitely, even at high abundance. To overcome many of the issues associated with targeted analysis, LC-HRMS has been more recently applied for screening and quantification of marine biotoxins, mainly to emerging ones [121]. 

### LC-MS Methods for Marine Biotoxins Determination in Food

Table S3 shows examples of LC-MS methods for the detection of marine biotoxins in foods worldwide during the last decade, 2011–2021. A total of 12 papers were selected, covering from 1 to 22 marine biotoxins in shellfish and derived products. The use of SPE column clean-up for the sample preparation, the HILIC in the LC separation and the QqQ, (MRM mode) in MS detection are the most used approaches in the selected papers.

Three papers focused on the use of LC-MS for the analysis of domoic acid in shellfish. In the first paper, an automated method based on the use of LC–MS/MS for the determination of domoic acid in shellfish was described by Regueiro et al. [122]. For the first time, the use of an online SPE based on weak anion exchange sorbent was proposed and favored a selective clean-up of shellfish extracts, as well as improved sensitivity of the method. The validated method was applied to the analysis of 12 samples including scallop, mussels, common cockle, Manila clam, oyster, razor clam and Macha. An LC-MS/MS method for the sensitive determination of domoic acid in mussel tissue was also reported by Beach et al. [123]. The method was based on SLE followed by strong anion exchange (SAX) SPE columns and a rapid and inexpensive derivatization step with dansyl chloride to form the dansyl derivative of domoic acid. The quantitative performances of the method were evaluated by the analysis of mussel tissue certified reference materials (CRMs) and results were in agreement with certified values. The last paper reported the optimization of an UHPLC-MS/MS analysis for the determination of domoic acid in mussel tissue [124]. After a methanol/water-based extraction, the extract was purified through a self-assembly IAC, which allowed an accurate and sensitive quantification. A validated method, having performance values that fulfilled the criteria reported in Codex Standard 292-2008 [125], was applied to the analysis of 59 Chinese real samples, with zhikong scallop being the most contaminated matrix. 

Blay et al. [120] described an LC-HRMS method, using Orbitrap technology, for the screening of 22 marine biotoxins, including 10 lipophilic and 12 hydrophilic ones, in mussel tissues using two different modes of chromatographic separation (i.e., reversed phase and HILIC, respectively). This kind of approach was suitable to be applied to other toxins or toxin analogues by expanding the target list of analyte masses during data processing. A multianalyte method was described by McCarron et al. [126] for monitoring and confirmation of domoic acid and another 12 lipophilic shellfish toxins using LC-MS/MS analysis in selected reaction monitoring (SRM) mode. The method, showing improved sensitivity and reduced matrix effect, was validated *in house* also using CRMs. Optionally, the use of an information-dependent acquisition approach in combination with the developed method provided an extra level of confirmation by library searching of product ion spectra also including unknown and unexpected isomeric analogues. The proposed method was applied to toxic mussel samples collected from western Canada in 2011, following an emergency, and highlighted the presence of high levels of dinophysistonin-1.

A single-laboratory validation of a HILIC-MS/MS method was described by Turner et al. [127] for the determination of 14 different paralytic shellfish toxins in 12 different species of bivalve shellfish (including a range of different mussel, oyster, clam, and scallop species) from both the UK and New Zealand. Prior to chromatographic separation, sample extracts were subjected to a clean-up using carbon SPE. The method was fully validated, and method performance characteristics were assessed, including trueness, ruggedness and uncertainty. The method showed, also, an excellent correlation by comparison with the reference method [128] for the analysis of 1141 shellfish tissues. Finally, the acceptability of the method was confirmed through successful participation in two separate proficiency testing studies.

Zhang et al. [129] described the development and validation of a new selective and sensitive method for the determination of tetrodotoxin in mussels tissue by a self-assembled IAC and UPLC-MS/MS analysis. The method was validated *in house* and applied to the analysis of 100 marine organisms including puffer fish, shrimps, crabs, clam and horseshoe crab collected from Chinese local markets with about 10% of samples contaminated with tetrodotoxin.

The development of a HILIC-MS/MS method for the determination of 14 paralytic shellfish toxins in oysters, greenshell mussels and dinoflagellate cultures was described by Thomas et al. [130]. The method was validated on two different LC-MS/MS systems, i.e., QTRAP 4000, in combination with OASIS HLB SPE clean-up, or QTRAP 5500 without any clean-up step, by assessing selectivity, linearity, LOD/LOQ, accuracy, precision and robustness. The two approaches were used to characterize a new CRM mussel tissue matrix that can be useful for the standardization and performance assessment of upcoming methods for paralytic shellfish toxins analysis.

Another study aimed to develop and validate a HILIC-MS/MS method for the detection of eight paralytic shellfish poisoning toxins in shellfish was reported by Yang et al. [131]. For the optimization of sample treatment, six different acidified extraction mixtures, and five different sorbents or sorbent mixtures for d-SPE preparation, were compared. The selected conditions included the use of acidified acetonitrile/water as extraction solvent and C18 silica and acid alumina as sorbent mixture. The method was validated according to the EU guidelines [17] and applied to the determination of saxitoxin, neosaxitoxin, gonyautoxins and the N-sulfo carbamoyl toxins C1 and C2 in 30 mussel samples of clam and scallops tissues from China, with 36.7% of them being positives to targeted toxins. 

Sibat et al. [132] described a comparative study between LC-MS/MS and LC-HRMS analysis for the detection of Pacific-ciguatoxin 1B and Pacific-ciguatoxin 3C in sea urchin, trochus shell, parrotfish and grouper fish. The sample preparation consisted of a solid liquid extraction with acetone, defatting with n-hexane and final purification on two SPE columns, i.e., Florisil^®^ and C18 cartridges. Then, three different chromatographic conditions (i.e., LC conditions, ion choice and acquisition modes) were compared for both LC-MS/MS and LC-HRMS analysis in order to investigate the selectivity of the targeted compounds. Although LC-MS/MS permitted an optimal sensitivity in agreement with EFSA and US guidelines, LC-HRMS allowed the identity confirmation of Pacific-ciguatoxins analogues.

Recently, a screening method for detection of anatoxin-a, cylindrospermopsin, saxitoxin, nodularin and microcystins in fish tissue was described by Haddad et al. [133]. Anatoxin-a, cylindrospermopsin and saxitoxin were analyzed by HILIC–MS/MS using for the first time a zwitterionic hydrophilic interaction LC column, while nodularin and microcystins were analyzed by RPLC-MS/MS. A SAX cartridge was used for SPE purification after SLE. The validated method was applied to fish tissues collected in Texas (USA), however no contaminated samples were found.

Recently, an ultra-performance HILIC–MS/MS was used for the screening of a total of 17 toxins, including paralytic shellfish toxins and tetrodotoxins, in bivalve molluscan species (mussels, oysters, cockles) collected in Sweden [134]. Similarly, to other studies, the sample preparation was carried out by solid liquid extraction and amorphous graphitized polymer carbon SPE purification, using in this specific case an automated system. The method was validated in house, obtaining LODs and LOQs that were far below the regulatory action limits [10,11]. 

## 5. Glycoalkaloids

Glycoalkaloids (GAs) are natural toxic secondary metabolites commonly found in the plants of the *Solanaceae* family, which contribute to plant resistance against pests and pathogens. Within *Solanacee* plants, the highest levels of GAs are found in potatoes, eggplants and tomatoes, even though the glycoalkaloids of most relevance to food safety, specifically α-solanine and α-chaconine, are those occurring in the potato (i.e., tubers, peel, sprouts, berries, leaves and blossoms). Several factors including mechanical damage to the plant, adverse storage (i.e., low temperature and bright light) and processing conditions may produce a significant increase of GAs content in potatoes [12]. In humans, acute toxic effects of potato GAs due to α-solanine and α-chaconine ingestion at low dose include gastrointestinal symptoms such as nausea, vomiting and diarrhea, while at higher doses more severe symptoms have been observed such as paralysis, neurological disorders, cardiac failure and coma [135]. However, GAs are not only known for their toxicity, but also for their health properties. Indeed, it has been reported that GAs possesses anticancer, anticholesterol, and anti-inflammatory properties. Various studies have shown the inhibitory effect of GAs on the growth of cancer cells originating from human skin, liver, prostate, breast and colon [136,137]. The EFSA Contam Panel identified an intake level of 1 mg/kg bw per day of total potato GAs/kg as a reference point for the risk characterization following acute exposure. Furthermore, exposure to 3–6 mg/kg bw per day of total potato GAs is considered to be potentially lethal for humans [12]. To date, no maximum levels for GAs in food or feed have been established at EU level, even though some European countries (i.e., Hungary, Finland, Sweden, Denmark, the Netherlands, Austria, Germany) have national legislation or recommendations on the maximum limits of total GAs, mainly in potato and potato products [12].

The determination of GAs can be carried out by using different confirmatory methods based on GC-MS, HPLC coupled with UV-vis detector or with MS or MS/MS. The use of matrix-assisted laser desorption/ionization time of flight mass spectrometry (MALDI-TOF MS) has been also reported. Enzyme-linked immunosorbent assay (ELISA) methods have been also described for GAs screening in food commodities [12]. 

Nowadays, HPLC methods have replaced GC methods; in particular, LC-MS based methods are increasing their popularity in the analysis of GAs due to their higher selectivity and sensitivity compared to traditional detectors. Methods based on LC-MS/MS are commonly used for targeted analysis of GAs, while methods using LC-HRMS, mainly with Orbitrap technology, are more frequently used for metabolomic studies and to detect unknown compounds. To reduce interferences of complex matrices efficient sample pretreatment methods including ultrasonic extraction, pressurized liquid extraction and SPE have been used [12,138]. 

### LC-MS Methods for Glycoalkaloids Determination in Food

Table S4 shows examples of published LC-MS methods for the detection of glycoalkaloids in foods worldwide during the last decade 2011–2021. A total of seven papers were selected covering from 2 to 19 GAs mainly in potato plants and derived products. 

In the first paper, Caprioli et al. [139] described a method based on liquid chromatography–hybrid linear ion trap–high-resolution mass spectrometry (LTQ-Orbitrap) for the determination of the GAs, α-solanine and α-chaconine, and their aglycons, demissidine and solasodine, in potato samples. Samples were extracted with methanol and purified by SPE using C18 cartridges before LC-MS analysis. The *in house* validated method was applied to screen 10 Irish potatoe samples, also evaluating if the cooking procedure affected the GAs content. Levels of GAs were higher in the skin than in the flesh, with concentrations lower in fried potatoes than those in boiled ones.

Three papers describing the use of LC-MS based methods for the determination of α-solanine and α-chaconine in potato and derived products are described in the present review. Firstly, Liu et al. [140] developed a method based on QuEChERS extraction and UPLC-MS/MS analysis for the quantification of the two GAs in commercial potato crisps. Linearity, sensitivity, accuracy and precision values were evaluated in the method validation study. The analysis of 20 commercial potato crisps showed that all samples were contaminated with targeted toxins, and levels of α-chaconine were higher than those of α-solanine. Subsequently, a UHPLC-MS/MS method for the determination of these two GAs was developed by Nie and Guo [141]. Samples were directly extracted using acetic acid aqueous solution. The method was validated according to the USFDA guidelines [142] for bioanalytical methods and showed accordance with the recommended performance criteria. The application of the method to potato samples of two different cultivars highlighted that the targeted glycoalkaloids were higher in the skin than those in the flesh and increased during storage under natural indoor conditions. Recently, for the first time a selective electromembrane extraction (EME) in combination with LC-MS/MS was proposed for the determination of α-solanine and α-chaconine in different potato tissues [143]. Several parameters of EME were optimized in terms of solvent type, extraction voltage, extraction time and the chemical composition of sample and acceptor solution. The method was validated according to the EURACHEM guidelines [73] and applied to the analysis of Chinese fresh potato peel, sprouted potato peel and sprouted potato tuber samples, with the latest showing the highest levels of targeted toxins. The EME approach showed great potential for extraction and purification of toxic compounds in complex plant tissues.

Lelario et al. [144] described an innovative approach based on the use of LC in combination with electrospray ionization Fourier transform ion cyclotron resonance mass spectrometry (LC-FTIR-MS) for the determination of 19 GAs in eggplant. The method was compared to the infrared multiphoton dissociation which is widely used as the method to produce a larger number of fragment ions. The proposed method was applied to the analysis of two varieties of eggplant berries grown in the Mediterranean area.

Another method was proposed by Lyu et al. [145] for the rapid screening of six glycoalkaloids in *Solanum scabrum* and *S. nigrum* berries from Kenya and US using UHPLC-MS/MS. Specifically, for the first time, the use of in-source fragmentation prior to the MS/MS analysis was applied as pseudo-MS to transform the GAs glycosides into the relevant aglycones. This approach permitted overcoming analytical issues due to the complexity and diversity of glycosides, as well as the limited availability of reference standards. 

Finally, a study reported the development of an LC-ESI-MS method for the quantification of α-solanine, its aglycon form solanidine, and the α-chaconine in potato protein isolates [146]. After an extraction with acetic acid aqueous solution, sample extracts were purified by HLB Oasis cartridges. The method was *in house* validated and applied to seven different protein isolates of relevance to the food industry to investigate the role of washing with water on GAs removal, and the effect of storage time on GAs level in protein from potato tubers following harvest. The results showed that total GAs increased during the storage of the potatoes.

## 6. Furocoumarins

Furocoumarins are natural toxins found mainly in edible plants belonging to the *Rutaceae* (e.g., grapefruit, lemon and lime), *Leguminosae* (e.g., soybean, beans, peanuts) and *Umbelliferae* (e.g., parsnip, parsley, celery, carrots) families. Furocoumarins have phototoxic properties and are produced in response to stress as defense against viruses, bacteria, fungi, insects and animals and are classified as natural pesticides [2]. On plants, in the presence of UV light, furocoumarins react with DNA of the predators by disrupting its replication and increasing their mortality. The three most phototoxic furocoumarins are psoralen, 5-methoxypsoralen and 8-methoxypsoralen. In humans, these toxins cause significant photodermatitis when skin is exposed to sunlight, leading to the development of blistered and burned skin. Furthermore, furocoumarins show mutagenic and carcinogenic activities in skin cells, and they may increase skin cancer risk [147,148]. Despite their toxic effects, some reports suggested that furocoumarins may potentially be used as therapeutic agents with anti-cancerogenic properties. Further studies are needed to evaluate their potential beneficial effect [148]. To date, at the EU level, no limits have been established for these furocoumarins in food yet. Several methods have been employed for the determination of furocoumarins in foods. The most common ones are based on HPLC with UV/DAD detectors. However, none of these methods allow for the identification and quantification of the complete set of toxins, including their isomeric forms, by guaranteeing high selectivity, sensitivity and short time of analysis [125]. UHPLC has proven to be a more efficient chromatographic separation tool, which not only obtains higher analytical sensitivity and better peak shapes, but also gives a greater resolution within a shorter retention time. The LC–MS/MS and LC-HRMS technologies are well known to be the best approaches in terms of selectivity and sensitivity compared to conventional LC methods, also allowing a chromatographic separation of isomers, therefore they can be considered suitable for the quantitative determination of furocoumarins in food.

### LC-MS Methods for Furocoumarins Determination in Food

Table S5 shows examples of published LC-MS methods for the detection of furocoumarins in foods worldwide during the last decade, 2011–2021. A total of six papers were selected covering from 7 to 21 furocoumarins mainly in citrus, grapefruit and relevant juices. Although some of these selected papers also reported the analysis of coumarins and polymethoxyflavones, or both, they have been included in this section because they are considered analytes belonging to the same class of compounds. 

A rapid UHPLC-MS method was described for the determination and quantitation of 21 furocoumarins and 6 coumarins in citrus peel (albedo and flavedo) after extraction with a methanol/water mixture [149]. The optimization of the UHPLC chromatographic conditions allowed the separation of the isomers of each furanocoumarin. The validated method was applied to the analysis of targeted furocoumarins and coumarins in citrus peel extracts from sweet orange, lemon, grapefruit, bergamot, pomelo, and clementine collected from France. Clementine and orange were characterized by low amounts of furocoumarins and coumarins as compared to the other citrus fruits. 

In another paper, the use of an LC-MS/MS method for simultaneous determination of nine furocoumarin and two coumarins of *Radix Angelicae Pubescentis* (RAP) and its related preparations (herbal, capsule and pill) was described for the first time by Li et al. [150]. The optimized sample preparation conditions were 70% methanol with ultrasonic assisted extraction (20 min). The developed method was applied to the analysis of four batches of RAP and seven related preparations (including one capsule and six pills) collected from different regions of China. Furocoumarin and coumarin content in the investigated samples did not exceed the minimum standards of Pharmacopoeia of the People’s Republic of China, except for psoralen in pills.

Two papers described the development of UPLC-MS/MS methods for the determination of bergaptol, psoralen, 8-methoxypsoralen, bergapten, 6′7′-dihydroxybergamottin, epoxybergamottin and bergamottin in American food by UPLC-MS/MS and using QuEChERS method for furocoumarins extraction. Firstly, Lee et al. [151] applied the in-house developed method to the analysis of the seven furanocoumarins in whole grapefruit, flesh, peel and grapefruit juice collected in Connecticut U.S. Bergamottin and 6′7′-dihydroxybergamottin were the main furocoumarins in grapefruit flesh and juice. In the second paper, Melough et al. [152] identified and quantified the same seven furocoumarins in 29 popularly consumed foods and beverages, belonging to the categories of citrus fruit and juices, figs, vegetables, herbs and spices, with the aim to create a database useful for a more accurate assessment of dietary furocoumarin exposure. The LODs and LOQs values and recovery of the *in house* validated method were suitable for the extraction of furocoumarins from the investigated samples. Specifically, all toxins were detected in 25 foods with fresh parsley containing the highest content of bergamottin, followed by bergapten and 6′7′-dihydroxybergamottin. The described study enabled a more accurate estimation of dietary furocoumarin exposure and was useful for epidemiological works investigating the relationships between furocoumarin intake and health outcomes. Furthermore, the authors compiled a database for furocoumarins representing the most comprehensive information on these toxins in popular foods and beverages available. 

The use of UHPLC-MS/MS was applied to the analysis, for the first time, of 15 furocoumarins, 8 coumarins and 7 phenolic acid esters in *Notopterygii Rhizoma et Radix* (NRR), an important constituent of traditional Chinese medicine [153]. For optimizing sample extraction and sample preparation, different extraction modes, extraction solvent, and extraction time were compared. The best candidates were ultrasonic extraction with methanol for 30 min. The validated method was successfully applied to the analysis of targeted active components in 10 batches of NRR samples collected from different Chinese regions. The two furocoumarins, isoimperatorin and 5-dehydronotopterol, together with another active component, exhibited the highest contents in the NRR; levels of bergapten, bergamottin and anhydronotopoloxide were at medium level, while the contents of the other analytes were relatively low or even not detected in individual samples.

Finally, Arigò et al. [154] reported the use of LC-MS/MS combined with the linear retention index system as an innovative analytical strategy for the characterization of 35 oxygen heterocyclic compounds that include furocoumarins, coumarins and polymethoxyflavones, in Italian citrus beverages. In that study, 19 furocoumarins were considered. The use of an MS/MS library and the LRI database guaranteed a reliable identification of targeted compounds in the citrus beverage, i.e., bergamot alcohol beverage, lemon juice, bergamot juice, earl grey tea, citrus infusion, lemon marmalade and homemade limoncello. Juice samples were only centrifuged, while marmalade and all the other samples were subjected to SLE and LLE, respectively, with ethyl acetate. Bergamot juice was the beverage with the highest amount of targeted compounds, mostly represented by bergamottin and bergapten.

## 7. Cyanogenic Glycosides

Cyanogenic glycosides (CNGs) are secondary plant metabolites present in more than 2600 species and are produced as a chemical defense response to herbivores and pathogens after tissue damage. Indeed, CNGs are hydrolyzed to cyanohydrins and hydrocyanic acid (HCN) upon contact with plant endogenous β-glycosidase following maceration or wounding of the plant, or by gut microflora, following ingestion of the plant material [155,156]. The most common families containing CNGs are *Fabaceae*, *Rosaceae*, *Leguminosae*, *Linaceae* and *Compositae*, while linamarin, linustatin, neolinustatin, lotaustralin, taxiphyllin, amygdalin, dhurrin and prunasin are the main CNGs that have received considerable attention from EFSA, due to their frequent occurrence in agri-food products [24]. CNGs are mainly found in fruits, such as almonds, apples, peaches, cherries, plums, bamboo shoots and apricots, vegetables, such as cassava plants and beans, and to a lesser extent in cereals, such as sorghum [157]. Other food products that may contain cyanogenic glycosides include some food ingredients with flavoring properties such as ground almonds powder or paste, marzipan, stone fruit, and alcoholic drinks made from stone fruits [158]. These foods therefore represent potential sources of HCN. Indeed, toxic levels of CNGs are estimated in terms of the quantity of HCN generated following hydrolysis, which differs considerably in vivo owing to many factors, including the structure of molecules and the quantity and type of bacteria [156,159]. Acute and subacute effects of exposure to cyanide are nausea, vomiting, headaches, abdominal cramps, muscle weakness, dizziness, convulsions, cardiac arrest, mental confusion, circulatory and respiratory failure, coma and in extreme cases death [160]. Moreover, CNGs can cause several chronic diseases mainly affecting the central nervous system and have drawn attention in international health risk assessment studies [24,161,162]. However, CNG metabolism remains unclear and considering that many foods are cyanogenic, an assessment of dietary safety is necessary. Based on EFSA risk assessment on apricot kernels [163] a maximum level of HCN in apricot seeds placed on the market, both whole and ground or split or ground, has been established at the European level [13]. Furthermore, the EC Regulation No. 1334/2008 provides maximum levels of certain substances naturally present in flavorings and food ingredients with flavoring properties, including HCN [14].

Quantification of CNGs in plant-based food can be made directly or indirectly. Specifically, direct methods are based on determining the intact glycoside in the sample and the few available methods are mainly based on HPLC analysis combined with UV-DAD or MS detection, with the latter ones improving sensitivity and selectivity. Less frequently, GC-MS methods, producing acceptable results in terms of high resolving power and automation, and ELISA assays, have been applied to quantify CNGs [24]. The indirect method of CNGs determination is based on the measurement of HCN, after acid or enzyme hydrolysis, by colorimetric, spectrophotometric or chromatographic methods. Although colorimetric determination is as sensitive as HPLC detection of CNGs, these methods cannot identify and quantify the specific CNGs because they are based on the estimation of total cyanide evolution rather than the detection of its source. Moreover, as different CNG enzymatic hydrolysis needs separate ß-glucosidases, the variability in the concentrations of these enzymes in seeds can increase analysis variability [24,157,164]. To evaluate the content of total cyanide in food, feed and derived products, standard methods have been set [165,166], while no standard methods are available for CNGs quantification in food yet. As more occurrence and consumption data for CNGs and cyanide in raw and processed foods are needed, the availability of validated methods, or new analytical techniques for their quantification is highly demanded. Furthermore, investigations on the variation of hydrolytic enzymes in food crops and the potential identification of cultivars with relatively low content of CNGs or hydrolytic enzymes are needed [24].

### LC-MS Methods for Cyanogenic Glycosides Determination in Food

Table S6 shows examples of published LC-MS methods for the direct detection of CNGs in foods during the last decade, 2011–2021. A total of nine papers were selected covering from one to eight CNGs in several plant foods, mainly including almonds and cassava samples. Two of them also determined the total amount of free and/or the total cyanide. 

Three papers described the use of LC-MS-based methods for the determination of amygdalin in almonds. In general, amygdalin is used as an indicator of the bitterness of almonds. Bitter almonds contain high levels of amygdalin which provides a characteristic cyanide aroma with moisture; non-bitter varieties contain trace levels of amygdalin and present nutty flavors. Finally, semi-bitter almonds have a “marzipan-like” taste. Specifically, Toomey et al. [167] described the application of LC-ITMS to the determination of amygdalin almonds associated with consumer complaints in California, after a simple SLE and dilution. Furthermore, the UV-Vis spectrophotometric determination was used for the determination of total cyanide. Results confirmed that the occurrence of amygdalin levels correlated with cyanide levels, as well as to the bitterness of almonds. Then, Lee et al. [168] applied the use of UHPLC-MS/MS for the quantification of amygdalin in non-bitter, semi-bitter and bitter almonds. The addition of acetic acid to the methanol/water extract prior to SPE clean-up through Hypersep C18 prevented the conversion of amygdalin to neoamygdalin. Amygdalin concentrations were determined in 10 commercial Californian varieties of non-bitter (sweet), semi-bitter and bitter almonds. The method was able to distinguish between non-bitter and bitter varieties. Furthermore, it was observed that the amygdalin concentration in commercial varieties was also related to the growing region. Finally, the identification and characterization of amygdalin, and its isomers neoamygdalin and amygdalin amide, in different processed bitter almonds was reported for the first time by Xu et al. [169] using HPLC-MS/MS analysis after an ultrasonic extraction with a methanol/water mixture. Specifically, raw, stir-fried and scalded bitter almonds from three producing areas in China were collected and analyzed. The quantification of amygdalin was achieved by HPLC-DAD. Amygdalin, neoamygdalin and amygdalin amide were identified in the different processed bitter almonds with no significant difference in the relative contents of total amygdalin isomers. 

The other two papers described the determination of linustatin and neolinustatin in flaxseeds. The first application was reported by Wang et al. [170], which used UHPLC-MS in SRM mode, after defatting, alkaline treatment and LLE, for the determination of these molecules in flaxseed products collected in China. The method also allowed the determination of the content of the beneficial constituent secoisolariciresinol diglucoside. A new three-step preparation method consisting of aqueous/methanol extraction of defatting flaxseeds, followed by alkaline extraction and then by hydrolysis was described for the first time. The method was validated according to ICH guidelines [72] to determine sensitivity, linearity, stability, precision, specificity and accuracy and successfully applied to the analysis of linustatin, neolinustatin and secoisolariciresinol diglucoside in non-coated flaxseeds. 

Gunasekera et al. [171] reported a rapid method to identify and quantify linamarin in cassava extracts by LC-MS, in SRM mode. A simple acidic methanol extraction provided the best extraction efficiency compared to extraction only with methanol or water at room temperature or at 80 °C, or alternatively with cryocooling extraction. The LC-MS method was applied to the analysis of freshly harvested tuber and leaves of cassava. 

Recently, Tanaka et al. [172] described the development of an UPLC-ESI-MS/MS method for the determination of amygdalin and prunasin in powdered loquat seeds, a product sold as a presumably health-promoting food in Japan. The measurement of total cyanide (by enzymatic treatment, steam distillation and colorimetric quantification) and free cyanide were also included. After ultra-sonication with water, a heating step at 100 °C was needed to inactivate the enzymatic hydrolysis by β-glucosidase that converted amygdalin into prunasin and glucose, and prunasin into hydrogen cyanide, glucose and benzaldehyde. The in-house validated method was applied to the analysis of 12 Japanese powdered loquat seeds. All samples contained more amygdalin than prunasin.

In another recent paper by Zhong et al. [173], the UHPLC-MS/MS was used for the simultaneous quantification of eight CNGs in agrifood products. After extraction with a methanol/water mixture, the extracts were purified through a Prime Hydrophilic-Lipophilic Balance cartridge. The method, validated according to the ICH guidelines [72], showed good sensitivity, precision and accuracy characteristics and was successfully applied to the analysis of cassava roots, bamboo shoots, linseed, apricot kernels, sorghum rice, almonds and lima bean.

Finally, an UHPLC-MS/MS method was developed and validated for the presence of amygdalin, dhurrin, prunasin and linamarin in American elderberry fruit [174]. The optimized sample preparation protocol provided the extraction with a mixture of methanol/water followed by a sample clean-up through C18 columns. Elderberry plant material samples (including fruits, tissues, seeds and juices) of different genotypes, and commercially processed elderberry juices, were analyzed for detection of CNGs. No quantifiable trace of the four targeted CNGs was detected in commercial elderberry juice, while traces of CNGs were detected in tissue samples.

## 8. Multiclass Methods

The LC-MS technology, either using triple quadrupole or high-resolution approaches, is the best and suitable approach in terms of selectivity and sensitivity for the simultaneous determination of multiclass analytes in such complex food matrices. Most of these methods use generic and rapid extraction protocols such as dilute and shoot and QuEChERS to cover as much as possible a wide range of analytes. 

Table S7 shows examples of published LC-MS methods for the multiclass detection of natural toxins alone or in combination with other toxic contaminants or bioactive compounds in foods during the last decade 2011–2021. A total of 13 papers were selected covering from one to four classes of natural toxins in food products, with mycotoxins and tropane alkaloids the more investigated classes. 

Wang et al. [170] proposed an UHPLC-MS method for the simultaneous determination of two cyanogenic glycosides, i.e., linustatin and neolinustatin, together with a lignan beneficial constituent, i.e., secoisolariciresinol, in defatted flaxseed. A methanol-based extraction was used before UHPLC-MS analysis in SRM mode. The reported method was successfully applied to monitor the levels of three targeted compounds in flaxseed powder during various processing methods.

In another paper, Lee et al. [175] described the development of a fast method using UPLC-ESI-HRMS for the qualitative and quantitative analysis of amygdalin together with other bioactive compounds in a medicinal herbal complex extract obtained from 23 Korean herbs traditionally used in oriental medicine. After extraction with ethanol/water, the extract was defatted with n-hexane and then hydrolyzed. The validated method was suitable to analyze for the first time simultaneously 18 bioactive compounds in the investigated matrix. 

A multi-class UHPLC-MS/MS method for the determination of beauvericin, enniatins A and B, enniatins A1 and B1, and cereulide (bacterial toxin) in corn, wheat, pasta and rice was reported by Decleer et al. [176]. A fast and simple liquid extraction procedure was used without any clean-up together with chromatographic separation of only 7 min provided recoveries higher than 84%. Validation performed according to Commission Decision 2002/657/EC [17] demonstrated the applicability of the method for the quantification of the targeted compounds. The validated method was applied to 57 naturally contaminated samples (rice, pasta and wheat) from several European and African countries. 

Three papers were focused on the development and validation of a multiclass method for the determination of mycotoxins and TAs in cereals and derived products. In the first paper, an LC-MS/MS method was developed and validated for the simultaneous determination of 20 ergot alkaloids (mycotoxins) and six TAs in 113 grain-based foods for infants and young children [177]. The proposed method was used to assess early variation in three sampling years (2011, 2012 and 2014) and results were shared with EFSA for their risk assessment studies. This study showed high concentration of TAs and ergot alkaloids in cereals for young children, as well as the advantages of the use of multiple toxins analytical methods for monitoring studies. A more recent paper described the extension of application of an LC-MS/MS method developed for the determination of the major mycotoxins to the determination of atropine and scopolamine in cereals [110]. Sample preparation was based on SLE and QuEChERS approach, while LC-MS/MS quantification was carried out by the isotopic dilution using labelled isotopomers as internal standards. The validation showed that the proposed method fulfilled the requirements of the SANTE/12682/2019 document [111] and then the method was successfully applied to the analysis of 95 samples collected from Asian and African countries. In the third paper, an LC-MS/MS method for the simultaneous quantification of 20 ergot alkaloids and six TAs in bread was reported by Versilovskis et al. [178]. A rapid and easy sample preparation protocol based on acidified/methanol extraction followed by an ultra-filtration was optimized. The validated method fulfilled the requirements of the SANTE/11813/2017 guidelines [103] and was applied to the analysis of 40 bread (based on wheat, multi-grain, rye and wheat-rye) samples collected in the Netherlands in 2014 and 2018. This study showed that ergot alkaloids were detected in a wide range of concentrations, thus highlighting the importance of monitoring their presence in bread. 

An LC-MS/MS-based multi-toxin method was applied to the quantitative determination of mycotoxins and CNGs in cassava samples [179]. The method, using the dilute--and-shoot approach, was validated for 106 mycotoxins and linamarin and lotaustralin using spiked samples, and was subsequently applied for the analysis of more than 300 analytes in 627 cassava samples collected from Tanzania and Rwanda. This study showed that although a broad range of mycotoxins were found, related concentrations exceeded 1 mg/kg in few cases while levels of CNGs were extremely high in few samples. 

Danezis et al. [180] reported a multi-residue HILIC-MS/MS method for the determination of aflatoxin B1, aflatoxin B2, fumonisin B1 and ochratoxin A in combination with pesticides, plant hormones and veterinary drugs, for a total of 28 analytes, in various food matrices. A rapid, easy and quick sample pretreatment was used for all tested matrices. The method was validated according to SANCO 12571/2013 document [181], showing good performance in terms of linearity, accuracy and sensitivity. Moreover, the combined and expanded uncertainty was also estimated for all tested analytes.

In a more recent study, the use of two-dimensional (2D) LC-MS/MS was reported for the first time for the determination of three classes of natural toxins, mycotoxins, tropane alkaloids and pyrrolizidine alkaloids, together with pesticides and growth regulators in oats and whole grains [182]. A simple acetonitrile/water extraction was used before the LC-MS/MS analysis with the matrix matched calibration curves approach. The validated method fulfilled requirements of the EURACHEM validation guide 2014 [74] and SANTE document 2015 [113], and can be considered a valuable tool for routine laboratory saving time and costs for monitoring targeted contaminants.

In the same year, Zhao et al. [183] proposed for the first time an UPLC-HRMS method for the determination of the two cyanogenic glycosides, linustatin and neolinustatin, and the lignan secoisolariciresinol diglucoside in flaxseed. A new procedure based on a double ultrasound-assisted extraction followed by a hydrolysis was optimized for the sample preparation. No purification step was used before UPLC-HRMS analysis. The method validated according to ICH guidelines [72] showed itself to be sensitive, selective, accurate and precise. A recent study carried out by Zhao et al. [184] was focused on the development of an UHPLC-MS/MS rapid method for the simultaneous determination of a wide range of secondary metabolites, such as furocoumarins, coumarins, flavonoids and phenolic acids in pummelo fruits. Although a rapid and generic ultrasonic assisted SLE was used, optimized chromatographic conditions and MRM mode acquisitions permitted the analysis of a total of 47 analytes including 13 groups of isomers. The validated method was successfully applied on pomelo fruits of two varieties collected in China.

Finally, another 2D-LC-MS/MS multiclass method was developed and validated for the determination of mycotoxins and TAs together with plant growth regulators and pesticides in cereals [185]. A rapid acetonitrile/water/formic acid extraction procedure was optimized before LC-MS/MS analysis. Validation of the proposed method was carried out according to regulations and guidelines set at EU level [17,52,111]. The validated method was applied to 36 cereal (wheat, barley, rice, oats, spelt and rye) samples showing the co-occurrence of 28 toxins and pesticides. 

## 9. Conclusions and Future Perspectives

Mass spectrometry-based methods (LC-MS) have become an essential tool for the monitoring of natural toxins as well as other chemical contaminants in food in order to ensure the safety of products, preserving consumer’s health. In this paper, we have reviewed the literature from 2011 to 2021 on the use of LC-MS for the detection of natural toxins in food. Specifically, the review focuses on the following six classes of natural toxins: mycotoxins, alkaloids, marine toxins, glycoalkaloids, cyanogenic glycosides and furocoumarins. The highest number of papers was collected for mycotoxins, therefore for this class the more restricted period from 2016 to 2021 was considered. Generally speaking, the most used sample preparation approaches included solid liquid extraction, followed by solid phase extraction column clean-up, or alternatively QuEChERS approaches. For the separation and detection mode, UHPLC is mainly used as an improved approach of chromatographic separation while MS/MS methodology is the current leading approach for detection, mainly for the analysis of known compounds. Specifically, the use of matrix-matched calibration curves and isotopically labelled standards allow compensating matrix effects, improving the accuracy of the methods. However, the use of LC-HRMS has become increasingly common for routine analysis. Indeed, thanks to the untargeted data acquisition, retrospective analysis can be carried out, allowing to screen and quantify also parent and unknown compounds as in the case of unexpected compounds. The LC-HRMS advance is mainly due to the capability of this technique to separate with high accuracy and sensitivity also closely related compounds, such as isomers. Hybrid instruments combining two different types of analyzers, mainly Q-TOF and Q-Orbitrap, are commonly applied to improve the selectivity and sensitivity of these analytical methods. Although the majority of available methods for the detection of natural toxins in food are in-house validated according to national or international protocols, much effort is still necessary to assess the suitability of published LC-MS methods for their standardization through interlaboratory validations according to international guidelines (such as AOAC/IUPAC, ISO, EU-DG SANTE). 

Recently, a clear trend in this sector is towards the development of new LC-MS methods capable of monitoring molecules showing different physicochemical properties, as in the case of natural toxins belonging to different classes, multicontaminants or multianalytes (e.g., bioactive compounds). The 2D-LC-MS/MS represents an alternative approach that is being successfully applied for the separation of multiclass analytes occurring in food complex matrices. Another emerging and interesting trend is represented by the application of HRMS using innovative untargeted metabolomics approaches to the screening of food samples without any prior information on the investigated analytes. As this approach is still challenging, efforts towards developing computational tools are necessary together with an appropriate implementation, validation and dissemination of databases for a wide range of analytes.

## Data Availability

Not applicable.

## Supplementary Materials

The following supporting information can be downloaded at: https://www.mdpi.com/article/10.3390/toxins14050328/s1, Table S1: Selected papers on the analysis of mycotoxins in food by liquid chromatography mass spectrometry; Table S2: Selected papers on the analysis of alkaloids in food by liquid chromatography mass spectrometry; Table S3: Selected papers on the analysis of marine toxins in food by liquid chromatography mass spectrometry; Table S4: Selected papers on the analysis of glycoalkaloids in food by liquid chromatography mass spectrometry; Table S5: Selected papers on the analysis of furocoumarins in food by liquid chromatography mass spectrometry; Table S6: Selected papers on the analysis of Cyanogenic glycosides in food by liquid chromatography mass spectrometry; Table S7: Selected papers on the analysis of multiclass of natural toxins in food by liquid chromatography mass spectrometry.
