# Supplementary material for: Overview of Recent Liquid Chromatography Mass Spectrometry-Based Methods for Natural Toxins Detection in Food Products
Source: Toxins (Basel). 2022 May 4;14(5):328. doi: 10.3390/toxins14050328 (PMC9143482; doi:10.3390/toxins14050328)
Supplement: Supplementary file 1 [file toxins-14-00328-s001.zip › toxins-1693556-supplementary.pdf]

Supplementary Materials: Overview of Recent Liquid Chromatography Mass Spectrometry-Based Methods for Natural Toxins Detection in Food Products

Annalisa De Girolamo Vincenzo Lippolis and Michelangelo Pascale

Table S1. Selected papers on the analysis of mycotoxins in food by liquid chromatography mass spectrometry.

Toxin	Matrix	Sample preparation	Analytical method	LOD	LOQ	Linearity range	Reference
Aflatoxin B1	Durum wheat	SLE	UPLC-MS/MS	2 μg/kg	3.5 μg/kg		[39]
Aflatoxin B2				2	3.5		
Aflatoxin G1				2	3.5		
Aflatoxin G2				2	3.5		
Ochratoxin A				2	3.5		
Enniatin A				5	10		
Enniatin A1				5	10		
Enniatin B				5	10		
Enniatin B1				5	10		
Sterigmatocystin				3	5		
Alternariol				3	7		
Alternariol methyl ether				8	15		
Tentoxin				2.5	5		
Beauvericin				10	15		
Diacetoxyscirenol				15	30		
T-2 toxin				7	15		
HT-2 toxin				30	70		
Neosolaniol				50	70		
Nivalenol				40	80		
Deoxynivalenol				30	70		
3-acetyl deoxynivalenol				15	30		
15-acetyl deoxynivalenol				30	45		
Zearalenone				30	70		
Fumonisin B1				35	70		

Fumonisin B2				70	80		
Fumonisin B3				70	80		
Nivalenol	Flour, corn grits, biscuits and pasta	SLE and SPE	LC-MS/MS	3.75 μg/kg	12.47 μg/kg	5-100 ng/mL	[41]
Deoxynivalenol				2.34	7.79	5-250	
HT-2 toxin				1.37	4.56	1-250	
T-2 toxin				0.38	1.25	1-100	
Zearalenone				0.75	2.50	1-100	
Aflatoxin B1	Palm, corn and sunflower oils	QuEChERS	LC-MS/MS	0.3 ng/g	1.0 ng/g	1-8 ng/g	[42]
Aflatoxin B2				0.1	0.4	0.4-20	
Aflatoxin G1				0.01	0.05	0.05-0.4	
Aflatoxin G2				0.2	1.0	1.0-7.0	
Ochratoxin A				3.0	10.0	10.0-80.0	
Zearalenone				1.5	5.0	5.0-40.0	
Deoxynivalenol				650	2000	2000-5500	
Deoxynivalenol	Wheat, corn and rice	QuEChERS and SPE	UPLC-MS/MS	5.0 μg/kg	15.0 μg/kg	5.0-500 μg/kg	[43]
3-acetyl deoxynivalenol				2.0	5.0	5.0-500	
15-acetyl deoxynivalenol				2.0	5.0	5.0-500	
Aflatoxin B1				0.03	0.1	0.1-50	
Aflatoxin B2				0.1	0.3	0.3-15	
Aflatoxin G1				0.1	0.3	0.1-50	
Aflatoxin G2				0.1	0.3	0.3-15	
Zearalenone				1.0	4.0	1.0-200	
Sterigmatocystin				0.1	0.4	0.4-200	
Ochratoxin A				0.1	0.4	0.4-200	
T-2 toxin				0.1	0.4	0.4-200	
HT-2 toxin				0.1	0.4	0.4-200	
Verruculogen				0.1	0.4	0.2-200	
Enniatin A				0.05	0.2	0.2-200	
Enniatin A1				0.05	0.2	0.2-200	
Enniatin B				0.05	0.2	0.1-200	
Enniatin B1				0.05	0.2	0.1-200	
Beauvericin				0.05	0.2	0.1-200	
Fusarenon-X				10.0	25.0	0.5-500	

Gliotoxin				5.0	10.0	5.0-200	
Neosolaniol				5.0	10.0	5.0-200	
Diacetoxyscirpenol				10.0	25.0	10.0-500	
Fumonisin B1				15.0	25.0	25.0-500	
Fumonisin B2				15.0	25.0	25.0-500	
Fumonisin B3				15.0	25.0	25.0-500	
Aflatoxin B1	Corn	SLE and d-SPE	UPLC-Q-TOF-MS	0.05 μg/kg	0.1 μg/kg	2.5-100 μg/L	[44]
Aflatoxin B2				0.05	0.1	2.5-100	
Aflatoxin G1				0.05	0.1	2.5-100	
Aflatoxin G2				0.05	0.1	2.5-100	
Fumonisin B1				5	15	10-1000	
Fumonisin B2				5	15	10-1000	
Fumonisin B3				5	15	10-1000	
Zearalenone				12	25	100-1000	
Deoxynivalenol				50	200	200-2000	
Aflatoxin B1	Radix Paeoniae Alba	QuEChERS and d-SPE	UHPLC-QqLIT-MS	0.03 μg/kg	0.25 μg/kg	0.25-100 μg/kg	[45]
Aflatoxin B2				0.08	0.20	0.20-0.30	
Aflatoxin G1				0.15	0.39	0.40-100	
Aflatoxin G2				0.10	0.30	0.30-30	
T-2 toxin				0.17	0.44	0.50-400	
Fumonisin B1				0.32	1.25	1.25-400	
Fumonisin B2				0.08	0.25	1-400	
Sterigmatocystin				0.08	0.25	0.5-400	
Penicillic acid				5.36	22.50	25-400	
Diacetoxyscirpenol				0.32	1.25	2.5-400	
Neosolaniol				0.32	3.86	5-400	
HT-2 toxin				2.35	3.35	5-400	
Ochratoxin A				0.20	0.5	0.5-400	
Deoxynivalenol				2.08	5.0	5-400	
Zearalenone				0.12	0.2	0.25-400	
Patulin				2.38	10.0	10-400	
3-acetyl deoxynivalenol				1.0	3.15	5-400	
15-acetyl deoxynivalenol				1.0	2.50	5-400	

Nivalenol				1.0	9.0	10-400	
Fusarenon-X				4.0	10.0	25-400	
Cyclopiazonic acid				0.07	0.5	0.5-400	
Alternariol	White wine, tomato juice, citrus juice	LLE and SPE	LC-MS/MS	0.12 μg/L	0.42 μg/L	0.20-0.09 μg/L	[46]
Alternariol methyl ether				0.10	0.36	0.05-0.09	
Altenuene/iso-altenuene				0.59	1.9	0.05-0.09	
Altenuisol				0.23	0.74	0.20-0.09	
AALtoxins TB1/TB2				0.33	1.1	0.05-0.09	
Tenuazonic acid				0.27	0.91	0.05-0.09	
Tentoxin				0.18	0.61	0.05-0.09	
Altertoxin I				0.22	0.74	0.10-0.09	
Altertoxin II				0.20	0.68	0.05-0.09	
Altenuic acid III				0.18	0.62	0.05-0.09	
Deoxynivalenol	Infant cereals (rice, barley, oats, mixed-grain)	SLE	UHPLC-MS/MS	0.5-10 ng/g	1-50 ng/g	0.1-100 ng/mL	[48]
Aflatoxin B1				0.1-0.5	0.5-1		
Aflatoxin B2				0.1-0.5	0.5-1		
Aflatoxin G1				0.1-0.5	0.5-1		
Aflatoxin G2				0.1-0.5	0.5-1		
Ochratoxin A				0.1	0.5		
Zearalenone				5	10		
Fumonisin B1				1-5	5-10		
Fumonisin B2				1	5		
T-2 toxin				0.05-0.1	0.1-0.5		
HT-2 toxin				0.5-1	1-5		
Aflatoxin B1	Cereal derived products (wheat flours, dry-pasta, baked foods, corn mils, breakfast cereals)	QuEChERS	LC-MS/MS	0.5 μg/kg	1 μg/kg	1.25-20 ng/mL	[49]
Aflatoxin B2				0.5	1	1.25-20	
Aflatoxin G1				0.5	1	1.25-20	
Aflatoxin G2				0.5	1	1.25-20	
T-2 toxin				1.3	2.5	1.125-50	
HT-2 toxin				1.3	2.5	3.125-50	
Fumonisin B1				100	200	20-120	
Fumonisin B2				100	200	20-120	
Deoxynivalenol		LLE and defatting	UHPLC-MS/MS		1.0 μg/kg		[50]

Aflatoxin G2					0.25		
Aflatoxin G1					0.1		
Aflatoxin B2					0.025		
Aflatoxin B1					0.1		
Fumonisin B1					0.1		
Fumonisin B2	Edible oils (soy bean, corn and rice bran oils)				0.1		
Fumonisin B3					0.1		
T-2 toxin					0.1		
Zearalenone					0.1		
Ochratoxin A					0.1		
Deepoxy-deoxynivalenol				0.758 ng/mL	3.0 µg/kg	3.03-30.30 ng/mL	
Aflatoxin G2				0.075	0.15	0.15-1.50	
Aflatoxin M1				0.025	0.05	0.05-0.50	
Aflatoxin G1				0.025	0.1	0.1-1.02	
Aflatoxin B2				0.020	0.04	0.04-0.40	
Aflatoxin B1				0.020	0.04	0.04-0.40	
HT-2 toxin				0.4	0.4	0.40-4.02	
Ochratoxin B	Cow milk	LLE and cleanup (salt)	LC-MS/MS	0.05	0.05	0.05-0.5	[52]
T-2 toxin				0.05	0.05	0.05-0.5	
Fumonisin B1				10.14	10.14	10.14-50.70	
Zearalenone				0.51	0.51	0.51-5.09	
Sterigmatocystin				0.125	0.5	0.50-5.02	
Ochratoxin A				0.2	0.2	0.2-1.00	
Fumonisin B3				0.625	2.5	2.5-25.0	
Fumonisin B2				2.5	2.5	2.5-25	
Nivalenol				12.4-18.1 µg/kg	37.6-46.1 µg/kg		
Deoxynivalenol				6-17.7	18.3-53.6		
3-acetyl deoxynivalenol				2.5-4.8	4.7-14.5		
Aflatoxin B1	Brown rice, millet, sorghum, corn and mixed cereals	SLE and IAC	LC-MS/MS	0.2-0.8	0.6-2.5	1.3-53 ng/mL	[53]
Aflatoxin B2				0.3-0.7	0.8-2.2		
Aflatoxin G1				0.1-0.5	0.4-1.4		
Aflatoxin G2				0.1-1.6	0.4-4.8		

Fumonisin B1				0.1-0.6	0.4-1.9		
Fumonisin B2				0.8-2.4	2.3-7.3		
HT-2 toxin				0.7-3.5	2.2-10.6		
T-2 toxin				4-15.3	12.2-46.4		
Zearalenone				1.9-6.3	5.8-19.2		
Ochratoxin A				0.4-2.3	1.9-7.0		
Aflatoxin B1				0.20-0.27 μg/kg	0.66-0.88 μg/kg	0.88-20 μg/kg (wheat)	
Aflatoxin B2				0.13-0.26	0.44-0.84	0.44-20	
Aflatoxin G1				0.18-0.27	0.58-0.89	0.89-20	
Aflatoxin G2				0.23-0.24	0.76-0.79	0.76-20	
Sterigmatocystin				0.14	0.46	0.46-20	
Ochratoxin A				0.52-0.58	1.72-1.90	1.90-200	
T-2 toxin				0.27-1.20	0.90-3.95	0.9-100	
HT-2 toxin				2.24-2.61	7.40-8.61	8.61-200	
Fumonisin B1				1.16-1.28	3.82-4.24	1.28-100	
Fumonisin B2				0.25-1.26	0.82-4.16	0.82-100	
Fumonisin B3				0.27	0.89-0.90	0.89-100	
Citrinin				2.36-2.63	7.78-8.69	8.69-150	
Roquefortin C	Wheat and corn	Salting-out LLE	UPLC-MS/MS	2.81-2.85	9.29-9.41	9.41-200	[54]
Zearalenone				2.70-2.95	8.92-9.74	8.92-200	
alpha-Zearalenol				2.46-2.48	8.12-8.19	8.12-200	
Deoxynivalenol				5.27-5.47	17.38-18.05	18.05-400	
Diacetoxyscirpenol				2.47-2.56	8.15-8.44	8.15-100	
3-acetydeoxynivalenol				4.81-5.73	15.87-18.90	15.87-400	
Fusarenon-X				5.57-5.81	18.37-19.16	18.37-400	
Neosolaniol				0.93-1.61	3.06-5.31	3.06-100	
Nivalenol				23.99-27.47	79.17-90.66	79.17-500	
Ergometrine				2.54-2.77	8.39-9.12	8.39-200	
Ergometrininine				2.32-2.97	7.64-9.79	9.79-200	
Ergotamine				2.70-2.98	8.91-9.84	8.91-200	
Ergotaminine				2.12-2.96	7.00-9.77	9.77-200	
Ergocronine				0.95-2.61	3.14-8.60	8.60-200	
Ergocroninine				1.98-2.83	6.53-9.34	6.53-200	

Ergokryptine				1.57-2.89	5.19-9.52	5.19-200	
Ergokriptinine				2.69-2.73	8.87-9.02	9.02-200	
Ergocronine				2.04-2.78	6.73-9.17	9.17-200	
Ergocroninine				1.96-2.69	6.46-8.86	8.86-200	
Ergosine				2.68-2.76	8.85-9.10	8.85-200	
Ergosininine				2.41-2.88	7.95-9.52	9.22-200	
Nivalenol<br>T-2 toxin<br>3-acetyl deoxynivalenol<br>15-acetyl deoxynivalenol<br>beta-Zearalenol<br>Zearalanone<br>Zearalenone<br>Neosolaniol<br>Deoxynivalenol<br>HT-2 toxin<br>Deepoxy-deoxynivalenol<br>alpha-Zearalenol<br>Diacetoxyscirpenol<br>T-2-3-glucoside<br>Deoxynivalenol-3 glucoside	Beer	LLE and SPE	UHPSFC-MS/MS				[55]
Fumonisin B1	Peach seed, milk powder, corn flour and beer	MA-d-μ-SPE	UHPLC-Q-TOF-MS	0.0030-0.0074 μg/kg	0.0089-0.022 μg/kg	0.0074-1.48 μg/mL	[56]
Aflatoxin B1				0.016-0.033	0.049-0.099	0.0635-2.54	
Ochratoxin A				0.016-0.022	0.047-0.066	0.034-1.36	
Ochratoxin B				0.0092-0.021	0.027-0.062	0.0088-1.76	
T-2 toxin				0.0022-0.0078	0.0065-0.023	0.018-3.6	
Zearalenone				0.0073-0.017	0.024-0.052	0.0098-1.96	
Sterigmatocystin	Corn	SLE and SPE	UPLC-MS/MS	1.0 μg/kg	2.0 μg/kg	2-500 ng/mL	[57]
Verruculogen				1.0	2.0	2-500	
Enniatin A				1.0	2.0	2-500	
Fusarenon-X				2.0	5.0	1-500	
Aflatoxin B1				0.5	1.0	1-500	

Aflatoxin B2				0.5	1.0	1-500	
Aflatoxin G1				0.5	1.0	1-500	
Aflatoxin G2				0.5	1.0	1-500	
Fumonisin B1				0.5	1.0	1-500	
Fumonisin B2				0.2	1.0	1-500	
Fumonisin B3				0.2	1.0	1-500	
3-acetyl deoxynivalenol				2.0	10.0	2-500	
15-acetyl deoxynivalenol				2.0	10.0	2-500	
Deoxynivalenol				1.0	2.0	2-500	
Ochratoxin A				0.5	1.0	1-500	
Zearalenone				0.3	1.0	1-500	
Ochratoxin A	Beer, apple juice, sunflower seed, walnut, nutmeg, tofu, red yeast rice food supplement, wheat flour, baby milk powder	QuEChERS	UPLC-MS/MS	0.1-1 µg/kg	0.3-1.9 µg/kg	from 0.25-100 to 1-3000 µg/kg	[58]
Citrinin				0.1-2.5	0.3-5.0	from 0.25-100 to 1-3000 µg/kg	
Deoxynivalenol	Buckwheat, corn and rice	SLE and two consecutives IAC	LC-MS/MS	12.1 µg/kg	36.8 µg/kg	36.8-3262.3 µg/kg	[59]
Aflatoxin B1				0.2	0.7	0.50-48.4	
Aflatoxin B2				0.2	0.5	0.17-16.0	
Aflatoxin G1				0.4	1.1	0.47-45.5	
Aflatoxin G2				0.1	0.3	0.14-13.8	
Fumonisin B1				39.2	118.7	118.7-5226.0	
Fumonisin B2				28.0	84.9	84.9-5205.2	
Fumonisin B3				24.6	74.5	74.5-5205.2	
T-2 toxin				1.0	2.9	2.9-326.6	
HT-2 toxin				2.2	6.6	5.0-331.2	
Zearalenone				14.7	44.5	20.0-3253.2	
Ochratoxin A				0.7	2.0	1.2-338.6	
3-acetyl deoxynivalenol	Peanut, almond and pistachio	QuEChERS and d-SPE	Nano-HPLC-HRMS	-	0.41-0.543 µg/kg	-	[60]
Aflatoxin B1					0.031-0.050		
Aflatoxin B2					0.041-0.048		
Aflatoxin G1					0.003-0.004		

Aflatoxin G2					0.003-0.005		
Aflatoxin M1					0.004		
alpha-Zearalenol					0.019-0.021		
Diacetoxyscirpenol					0.031-0.102		
Ergocornine 1					0.074-0.078		
Ergocornine 2					0.072-2		
Fumonisin B1					0.032-0.102		
Fumonisin B2					0.331-2		
Gliotoxin					0.34-0.442		
T-2 toxin					0.442-0.505		
HT-2 toxin					0.265-0.397		
Ochratoxin A					0.014-0.017		
Zearalenone					0.010-0.049		
Alternariol	Cherry tomato, leafy vegetables, strawberry and tomato	SLE and SPE	UHPLC-MS/MS	0.3 µg/L	1 µg/L	0.5-80 µg/L	[61]
Alternariol methyl ether				0.05	0.2	0.1-40	
Tenuazonic acid				0.3	1	0.5-80	
Tentoxin				0.1	0.4	0.2-40	
Ochratoxin A				0.5	1.5	0.7-80	
Patulin				3	10	7-200	
Deoxynivalenol				1.5	5	3.5-200	
Aflatoxin B1	Wheat and corn	Method 1: SLE and dilute-and-shoot Method 2: SLE and SPE	LC-MS/MS	0.5 µg/kg	1.6 µg/kg	1.6-80 µg/kg	[62]
Aflatoxin B2				0.3	1.0	1.0-20	
Aflatoxin G1				0.5	1.6	1.6-80	
Aflatoxin G2				0.3	1.0	1.0-20	
3-acetyl deoxynivalenol				3.0	10.0	10-2000	
15-acetyl deoxynivalenol				3.0	10.0	10-2000	
Deoxynivalenol				1.2	4.2	4.2-8400	
Deoxynivalenol-3 glucoside				3.0	10.0	10-1000	
Enniatin A				0.5	1.6	1.6-1620	
Enniatin A1				0.6	1.8	1.8-1800	
Enniatin B				0.4	1.4	1.4-1400	
Enniatin B1				0.5	1.5	1.5-1520	
Fumonisin B1				1.5	5.0	5.0-10000	

Fumonisin B2				0.6	2.0	2.0-2000	
Moniliformin				0.3	1.0	1.0-2000	
Nivalenol				3.0	10.0	10.0-2000	
Zearalenone				0.4	1.2	1.2-2400	
Aflatoxin B1	Korean soybean paste Doenjang	SLE and IAC	LC-MS/MS	0.08 μg/kg	0.26 μg/kg	0.5-20 μg/kg	[63]
Aflatoxin B2				0.14	0.43	0.5-20	
Aflatoxin G1				0.10	0.30	0.5-20	
Aflatoxin G2				0.15	0.45	0.5-20	
Ochratoxin A				0.06	0.17	25-1000	
Zearalenone				4.68	14.18	25-1000	
Zearalanone				1.66	5.03	25-1000	
alpha-Zearalenol				1.47	4.44	25-1000	
alpha-Zearalanol				2.29	6.94	25-1000	
beta-Zearalenol				1.72	5.23	25-1000	
beta-Zearalanol				2.13	6.45	25-1000	
T-2 toxin				2.55	7.73	25-1000	
HT-2 toxin				1.86	5.65	25-1000	
Nivalenol				3.21	9.74	25-1000	
Deoxynivalenol				4.37	13.24	25-1000	
3-acetyl deoxynivalenol				0.9	2.73	25-1000	
Deoxynivalenol-3 glucoside				2.72	8.24	25-1000	
Fumonisin B1				2.57	7.78	25-1000	
Fumonisin B2				2.16	6.56	25-1000	
Fumonisin B3				2.22	6.72	25-1000	
Nivalenol	Wheat, wheat flour, wheat crackers	SLE and SPE	LC-MS/MS	10 μg/kg			[64]
Deoxynivalenol				5			
3-acetyl deoxynivalenol				1.0			
15-acetyl deoxynivalenol				1.0			
T-2 toxin				0.5			
HT-2 toxin				0.5			
Zearalenone				0.5			
Aflatoxin B1	Dried fish, dried shrimp and dried mussel	UAE and cleanup (defatting)	LC-MS/MS	0.1 μg/kg	0.3 μg/kg	0.5-100 μg/kg	[66]
T-2 toxin				0.1	0.5	0.5-200	
Ochratoxin A				0.1	0.5	0.5-100	

Deoxynivalenol				1.0	3.0	1-500	
Aflatoxin B1	Corn	PHWE	LC-MS/MS	0.94 μg/kg	2.8 μg/kg	160-5000 μg/mL	[67]
Aflatoxin B2				0.08	0.3		
Aflatoxin G1				0.43	1.3		
Aflatoxin G2				1.7	5.1		
Alternariol methyl ether				2.4	7.2		
Fumonisin B1				2.3	6.8		
Fumonisin B2				4.3	13.0		
Fumonisin B3				0.1	0.2		
Ochratoxin A				0.4	1.3		
Ochratoxin B				0.1	0.3		
Sterigmatocystin				0.5	1.5		
T-2 toxin				16.0	47.3		
Zearalenone				1.6	4.9		
alpha-Zearalenol				41.0	123		
beta-Zearalenol				12.0	36.0		
Moniliformin	Malted barley and beer	QuEChERS	UPLC-QTOF-MS	5 μg/kg	15 μg/kg	15-3000 μg/kg (malt)	[68]
Deoxynivalenol				1.0-2.5	5-10	10-1000	
Fumonisin B1				15	50	50-500	
Aflatoxin B1				0.15	0.25	0.25-100	
Aflatoxin B2				0.05	0.25	0.25-50	
Aflatoxin G1				0.05	0.25	0.25-50	
Aflatoxin G2				0.05	0.25	0.25-50	
Ochratoxin A				0.05-0.10	0.125-0.25	0.125-20	
T-2 toxin				1.5	5	50-1500	
HT-2 toxin				2.0-2.5	5	50-2000	
Zearalenone				0.05-0.1	0.125-0.25	0.25-250	
Sterigmatocystin				5.0	12.5	12.5-1800	
Enniatin A				0.01	0.05	0.05-250	
Enniatin A1				0.01-0.5	0.05-0.125	0.125-250	
Enniatin B				0.01-0.5	0.05-2.5	2.5-250	
Enniatin B1				0.01	0.05	0.05-250	
Beauvericin				0.025	0.125	0.25-100	
Aflatoxin B1	Rice bran	SLE and DLLME	LC-MS/MS	0.5 ng/g	1.5 ng/g	-	[69]

Aflatoxin B2				0.5	1.5		
Aflatoxin G1				1.0	3.0		
Aflatoxin G2				1.0	3.0		
Sterigmatocystin				2.5	5.0		
T-2 toxin				2.5	5.0		
HT-2 toxin				2.5	5.0		
Zearalenone				2.5	5.0		
Diacetoxyscirpenol				2.5	5.0		
Fumonisin B1				50	150		
Fumonisin B2				25	75		
Ochratoxin A				1.25	3.75		
Aflatoxin B1	Traditional Chinese medicinal materials (Polygalae Radix, Coicis Semen and Eupolyphaga Steleophaga)	SLE and Multi-IAC	LC-MS/MS	0.007 ng/mL	0.02 ng/mL	0.05-25 ng/mL	[70]
Aflatoxin B2				0.007	0.02	0.10-10	
Aflatoxin G1				0.003	0.01	0.05-25	
Aflatoxin G2				0.03	0.1	0.30-15	
Deoxynivalenol				0.2	0.5	1.0-250	
Zearalenone				0.003	0.05	0.50-250	
Ochratoxin A				0.02-0.05	0.05	0.20-10	
T-2 toxin				0.04	0.12	0.50-250	
Fumonisin B1				0.08	0.25	0.40-1000	
Fumonisin B2				0.03	0.1	0.20-500	

Table S2. Selected papers on the analysis of alkaloids in food by liquid chromatography mass spectrometry.

Toxin	Matrix	Sample preparation	Analytical method	LOD	LOQ	Linearity range	Reference
Hyoscyamine (Atropine)<br>Scopolamine (Hyoscine)	Plant organs of Datura species	SLE	LC-MS	50 pg/mL<br>100	167 pg/mL<br>333	0.001-1 μg/mL<br>0.001-1	[88]
Crotaline<br>Lycopsamine<br>Jacobine<br>Retrorsine<br>Heliotrine<br>Trichodesmine<br>Otosenine<br>Seneciphylline<br>Senecionine<br>Echimidine<br>Senkirkine	Honey	SLE and SPE	LC-ITMS	0.0134 μg/mL<br>0.0182<br>0.0293<br>0.0250<br>0.0278<br>0.0305<br>0.0186<br>0.0198<br>0.0237<br>0.0230<br>0.0146	0.046 μg/ml<br>0.0608<br>0.0976<br>0.0833<br>0.0928<br>0.1018<br>0.0621<br>0.0660<br>0.0790<br>0.0765<br>0.0487	0.1-1.0 μg/mL	[89]
Lycopsamine<br>Senecionine<br>Senecionine N-oxyde<br>Heliosupine<br>Echimidine	Officinalis plant parts (roots, flowers, stems, leaves) and honey	SLE and SPE	LC-MS	1-2 ng/mL	1.43 ng/mL<br>1.38<br>1.58<br>1.89<br>1.21	up to 150 ng/mL	[90]
Anisodine<br>Scopolamine<br>Anisodamine<br>Atropine	Przewalskia tangutica Maxim. fruit extracts	SLE and MIP-SPE	LC-MS	1.00 μg/L<br>2.12<br>1.05<br>0.52	-<br>-<br>-<br>-	5-1000 μg/L<br>5-1000<br>5-1000<br>5-1000	[92]
Echimidine<br>Heliotrine<br>Lycopsamine<br>Retrorsine<br>Senecionine<br>Seneciphylline<br>Retrorsine N-oxyde	Honey	SLE and SPE	LC-MS/MS		0.25 μg/kg	0.25-50 μg/kg	[93]
Echimidine	Honey		LC-MS/MS	0.1 μg/kg	0.2 μg/kg	0-50 μg/kg	[94]

Intermedine + lycopsamine<br>Monocrotaline<br>Retrorsine<br>Retrorsine N-oxyde<br>Senecionine<br>Senecionine N-oxyde		SLE and dilute-and-shoot		0.1<br>0.3<br>0.1<br>1.0<br>0.1<br>0.2	0.2<br>0.5<br>0.2<br>1.5<br>0.2<br>0.5		
Atropine<br>Scopolamine	Buckwheat, soy, millet, chia	QuEChERS and d-SPE	UHPLC-HRMS	0.2 µg/kg<br>0.04		0.1-100 µg/L	[95]
Atropine<br>Scopolamine<br>Echimidine<br>Heliotropine<br>Intermedine<br>Lasiocarpine<br>Lycopsamine<br>Retrorsine<br>Senecionine<br>Seneciphylline<br>Senkirkine	Honey	QuEChERS and d-SPE	LC-HRMS	0.1 µg/kg<br>0.2<br>0.1<br>0.1<br>0.2<br>0.04<br>0.2<br>0.1<br>0.1<br>0.1<br>0.1	0.5 µg/kg<br>0.5<br>0.2<br>0.3<br>0.7<br>0.1<br>0.6<br>0.4<br>0.4<br>0.3<br>0.2	0-10.5 µg/kg	[85]
Echimidine, echimidine N-oxide, erucifoline, erucifoline N-oxide, europine (as hydrochloride), europine N-oxide, heliotrine, heliotrine N-oxide, intermedine, intermedine N-oxide, jacobine, jacobine N-oxide, lasiocarpine, lasiocarpine N-oxide, lycopsamine, opsamine N-oxide, monocrotaline, monocrotaline N-oxide, retrorsine, retrorsine	Cow milk, tea infusion, honey, cooked chicken, egg, cooked beef, barley flour, clove leave	USLE and SPE	UPLC-MS/MS	-	0.010-0.76 µg/kg	0.005-1.0 µg/L	[86]

Noxide, senecionine, senecionine N-oxide, seneciphylline, seneciphylline N-oxide, senecivernine, senecivernine N-oxide, senkirkine, trichodesmine							
Atropine<br>Scopolamine	Buckwheat organic foods (flour, pasta, crackers, plumcakes)	SLE and dilute-and-shoot	UHPLC–MS/MS	0.09 µg/kg<br>0.03	0.3 µg/kg<br>0.1	2.5-50 µg/kg<br>2.5-50	[98]
Intermedine<br>Lycopsamine<br>Jacobine<br>Retrorsine<br>Heliotrine<br>Seneciphylline<br>Senecionine<br>Echimidine<br>Senkirkine<br>Lasiocarpine	Honey	SLE and SPE	LC-MS		1.0 µg/kg	0-50 µg/kg	[100]
Echimidine<br>Echimidine N-oxide<br>Erucifoline<br>Erucifoline N-oxide<br>Europine<br>Europine N-oxide<br>Heliotrine<br>Heliotrine N-oxide<br>Indicine<br>Indicine N-oxide<br>Intermedine N-oxide<br>Intermedine<br>Jacobine	Honey	SLE and SPE	LC-HRMS	0.7 µg/kg<br>0.5<br>0.7<br>0.5<br>1.0<br>0.5<br>0.5<br>0.2<br>0.2<br>0.5<br>0.5<br>0.5<br>2.0	2.0 µg/kg<br>2.0<br>2.0<br>2.0<br>2.0<br>1.0<br>0.7<br>0.5<br>2.0<br>2.0<br>2.0<br>1.0<br>2.0	5-200 µg/kg	[102]

Jacobine N-oxide				1.0	5.0		
Lasiocarpine				0.5	5.0		
Lasiocarpine N-oxide				0.5	1.0		
Lycopsamine				0.2	0.7		
Lycopsamine N-oxide				0.7	0.7		
Monocrotaline				2.0	2.0		
Monocrotaline N-oxide				0.7	5.0		
Retrorsine				1.0	2.0		
Retrorsine N-oxyde				0.7	2.0		
Senecionine				0.7	2.0		
Senecionine N-oxyde				0.5	2.0		
Seneciphylline				0.7	2.0		
Seneciphylline N-oxide				0.5	2.0		
Senecivernine				0.5	1.0		
Senecivernine N-oxide				0.7	2.0		
Senkirkine				0.5	2.0		
Trichodesmine				3.0	0.7		
Erucifoline N-oxide				0.13 µg/kg	0.66 µg/kg		
Lasiocarpine				0.12	0.59		
Lycopsamine N-oxide				0.10	0.50		
Retrorsine				0.09	0.45		
Senecionine N-oxyde				0.11	0.53		
Seneciphylline	Gynura procumbens herb	SLE and SPE	LC- MS/MS	0.12	0.59	10-500 µg/kg	[104]
Senkirkine				0.13	0.66		
Senecivernine				0.09	0.89		
Seneciphylline N-oxide				0.11	0.55		
Heliotrine				0.17	0.85		
Retronecine				0.18	0.45		
Echimidine				0.54 µg/kg	1.07 µg/kg		
Intermedine				0.41	0.81		
Intermedine N-oxide	Honey	SLE and SPE	LC-HRMS	0.52	1.04	2-150 ng/mL	[105]
Lasiocarpine				0.22	0.45		
Lasiocarpine N-oxide				0.60	1.20		

Lycopsamine				0.28	0.56		
Retrorsine				0.34	0.67		
Retrorsine N-oxyde				0.64	1.27		
Senecionine				0.45	0.91		
Senecionine N-oxyde				0.42	0.84		
Seneciphylline				0.23	0.45		
Seneciphylline N-oxide				0.60	1.19		
Atropine	Animal derived products (porcine muscle, egg, milk)	QuEChERS and d-SPE	LC-MS/MS	0.8 μg/kg	2 μg/kg	2-12 μg/kg	[107]
Scopolamine				1.0	5	5-30	
Sparteine				0.4	1	1-6	
Atropine	Raw cereals (corn, oat, quinoa, soybean, wheat) and cereals-based products (infant cereals)	SLE and QuEChERS and defatting	UPLC-MS/MS	-	0.5 μg/kg	0.5-50 μg/kg	[108]
Scopolamine				--	0.5	0.5-50	
6-beta-hydroxytropinone	Sorghum, oregano, herbal tea	SLE and d-SPE	UHPLC-MS/MS HILIC–MS/MS*	-	1-10 μg/kg	0.5-1000 μg/kg (matrix-matched calibration curve)	[110]
Anisodamine				-	1.0		
Anisodine				-	1.0		
Apoatropine				-	1.0		
Aposcopolamine				-	1.0		
Atropine				-	0.5-2		
Convolamine				-	1.0-2.0		
Convolidine				-	1.0-2.0		
Convolvine				-	1.0		
Fillalbine				-	1.0		
Homatropine				-	1.0-5.0		
Hydroxymethyl-atropine				-	1.0-2.0		
Littorine				-	1.0-10.0		
Noratropine				-	0.5-2.0		
Nortropinone				-	1.0-5.0		
Norscopolamine				-	1.0		
Phenylacetoxytropane				-	0.5-1.0		
Pseudotropine				-	1.0-10.0		
Scopolamine				-	0.5-2.0		
Tropine				-	2.0-10.0		

Tropinone	-	2.0-10.0
Echimidine	-	0.5-1.0
Echinatine*	-	0.5-5.0
Erucifoline	-	1.0-5.0
Europine	-	1.0-5.0
Heliotrine	-	1.0
Indicine*	-	2.0-10.0
Intermedine*	-	0.5-10.0
Jacobine	-	1.0-5.0
Lasiocarpine	-	0.5-2.0
Lycopsamine*	-	2.0-10.0
Monocrotaline	-	1.0-5.0
Retronecine	-	2.0-10.0
Retrorsine	-	1.0-5.0
Senecionine	-	1.0-5.0
Seneciphylline	-	1.0-5.0
Senecivernine	-	1.0-5.0
Senkirkine	-	0.5-1.0
Trichodesmine	-	1.0-2.0
Echimidine N-oxide	-	1.0-2.0
Echinatine N-oxide*	-	1.0-5.0
Erucifoline N-oxide	-	1.0-5.0
Europine	-	1.0-10.0
Heliotrine N-oxide	-	1.0
Indicine N-oxide	-	2.0-5.0
Intermedine N-oxide	-	1.0-5.0
Jacobine N-oxide	-	1.0-5.0
Lasiocarpine N-oxide	-	1.0-5.0
Lycopsamine N-oxide*	-	1.0-5.0
Monocrotaline N-oxide	-	1.0-10.0
Retrorsine N-oxyde	-	1.0-10.0
Senecionine N-oxyde	-	1.0-10.0
Seneciphylline N-oxide	-	1.0-10.0
Senecivernine N-oxide	-	1.0-5.0

Atropine<br><br>Scopolamine	Black tea, peppermint, fennel, cereal products	SLE and dilute-and-shoot	LC-MS/MS	0.08-1.60 µg/kg<br><br>0.16-0.38	0.28-5.29 µg/kg<br><br>0.52-1.25	0.3-20 µg/kg (cereal products); 0.6-40 µg/kg tea and herbs	[111]
Echimidine<br>Echimidine N-oxide<br>Erucifolin<br>Erucifolin N-oxide<br>Europine<br>Europine N-oxide<br>Heliotrine<br>Heliotrine N-oxide<br>Indicine<br>Indicine N-oxide<br>Intermedine<br>Intermedine N-oxide<br>Jacobin<br>Jacobin N-oxide<br>Lasiocarpine<br>Lasiocarpine N-oxide<br>Lycopsamine<br>Lycopsamine N-oxide<br>Monocrotaline<br>Monocrotaline N-oxide<br>Retrorsine<br>Retrorsine N-oxyde<br>Senecionine<br>Senecionine N-oxyde<br>Seneciphylline<br>Seneciphylline N-oxide<br>Senecivernine<br>Senecivernine N-oxide<br>Senkirkine<br>Senkirkine N-oxide	Pepermint, chamomile, nettle, linden	SLE and ultrasonic assisted QuEChERS and d-SPE	LC-MS/MS		1.0 µg/kg	0.1-500 ng/mL	[112]

Atropine<br>Scopolamine	Honey	SLE	HILIC–MS/MS	0.002 µg/kg<br>0.003	0.01 µg/kg<br>0.01	0.02-2 µg/kg<br>0.02-2	[113]
Echimidine	Tussilago farfara and Lithospermi erythrorhzion	SLE	LC-MS/MS	0.1 µg/kg	0.2 µg/kg	1.0-40 ng/mL	[114]
Echimidine N-oxide				1.7	5.1	1.0-40	
Erucifoline				0.3	1.0	0.5-20	
Erucifoline N-oxide				0.2	0.6	0.5-20	
Europine				0.1	0.3	0.5-21	
Europine N-oxide				0.03	0.1	0.5-22	
Heliotrine				0.03	0.1	0.5-23	
Heliotrine N-oxide				0.1	0.3	0.5-24	
Intermedine				0.1	0.2	0.5-25	
Intermedine N-oxide				0.3	0.8	0.5-26	
Jacobine				3.5	10.4	2.0-80	
Jacobine N-oxide				0.1	0.3	0.5-20	
Lasiocarpine				0.1	0.3	2.0-80	
Lasiocarpine N-oxide				8.1	24.2	2.0-80	
Lycopsamine				0.1	0.3	0.5-20	
Lycopsamine N-oxide				0.4	1.1	1.0-40	
Monocrotaline				0.3	0.8	0.5-20	
Monocrotaline N-oxide				0.2	0.6	0.5-20	
Retrorsine				3.1	9.3	1.0-40	
Retrorsine N-oxyde				1.5	4.4	1.0-40	
Senecionine				-	-	2.0-200	
Senecionine N-oxyde				-	-	2.0-200	
Seneciphylline				0.5	1.6	0.5-20	
Seneciphylline N-oxide				0.5	1.6	0.5-20	
Senecivernine				3.0	9.0	1.0-40	
Senecivernine N-oxide				0.8	2.3	1.0-40	
Senkirkine				-	-	2.0-200	
Trichodesmine				0.3	0.9	1.0-40	

Table S3. Selected papers on the analysis of marine toxins in food by liquid chromatography mass spectrometry.

Toxin	Matrix	Sample preparation	Analytical method	LOD	LOQ	Linearity range	Reference
Domoic acid (DA)	Mussels tissues	SLE	LC-MS	3.4 μg/L	-	--	[121]
N-sulfocarbamoyl-C1 (C1)				14	-		
N-sulfocarbamoyl-C2 (C2)				11	-		
Gonyautoxin-1 (GTX1)				9.9	-		
Gonyautoxin-2 (GTX2)				7.5	-		
Gonyautoxin-3 (GTX3)				4.5	-		
Gonyautoxin-4 (GTX4)				6.7	-		
Gonyautoxin-5 (GTX5)				6.9	-		
Decarbamoylgonyautoxins-2 (dcGTX2)				7.2	-		
Decarbamoylgonyautoxins-3 (dcGTX3)				4.0	-		
Saxitoxin (STX)				6.0	-		
Neosaxitoxin (NEO)				8.1	-		
Gymnodimine (GYM)				0.041	-		
13-desmethyl spirolide C (SPX1)				0.054	-		
Yessotoxin (YTX)				5.1	-		
Okadaic acid (OA)				2.8	-		
Dinophysistotoxin-1 (DTX1)				2.0	-		
Dinophysistotoxin-2 (DTX2)				1.6	-		
Pectenotoxin-2 (PTX2)				0.10	-		
Azaspiracid-1 (AZA1)				0.052	-		
Azaspiracid-2 (AZA2)				0.064	-		
Azaspiracid-3 (AZA3)				0.062	-		
Domoic acid (DA)	Scallop, mussels, common cockle, Manila clam	SLE and online SPE	LC–MS/MS	0.23-0.32 μg/kg	-	0.05-100 ng/mL	[123]
Domoic acid (DA)	Mussels	SLE and hydrolyzation	LC-MS/MS	10 μg/kg	-	19-4300 ng/mL	[127]
Okadaic acid (OA)				18	-	2.3-58.7	
Dinophysistotoxin-1 (DTX1)				20	-	2.6-68.3	
Dinophysistotoxin-2 (DTX2)				19	-	2.0-51.9	
Azaspiracid-1 (AZA1)				1	-	0.2-34.0	

Azaspiracid-2 (AZA2)				2	-	0.5-11.1	
Azaspiracid-3 (AZA3)				1	-	0.1-8.9	
Yessotoxin (YTX)				35	-	7.1-62.0	
Homo-Yessotoxin (hYTX)				28	-	8.0-207.2	
Pectenotoxin-2 (PTX2)				0.5	-	0.4-82.7	
Gymnodimine (GYM)				1	-	0.2-35.0	
Spirolide-1 (SPX1)				1	-	0.2-34.2	
Pinnatoxin-G (PnTX-G)				0.5	-	0.2-37.9	
Domoic acid (DA)	Mussel tissue (CRM)	SLE and SPE	LC–MS/MS (after dansylation)	1.1 μg/kg	3.7 μg/kg	-	[124]
N-sulfocarbamoyl-C1 (C1)	Mussels, oysters, clam and scallops tissues	SLE and SPE	LC(HILIC)–MS/MS	5.6-14 nmol/kg	19-45 nmol/kg	5.7-227 nmol/L	[128]
N-sulfocarbamoyl-C2 (C2)				1.5-3.8	5-13	1.7-68	
Decarbamoylgonyautoxin-2 (dcGTX2)				5.4-15	18-50	5.7-228	
Decarbamoylgonyautoxin-3 (dcGTX3)				2.7-5	9-17	1.6-64	
Gonyautoxin-2 (GTX2)				0.32-7.4	1.1-2.5	5.7-228	
Gonyautoxin-3 (GTX3)				1.3-8.6	4.3-29	2.2-87	
Gonyautoxin-1 (GTX1)				3.8-11	13-36	3-121	
Gonyautoxin-4 (GTX4)				3.8-8.5	13-28	1.0-39	
Gonyautoxin-5 (GTX5)				2.2-7.5	7.3-25	3.3-130	
Decarbamoylsaxitoxin (dcSTX)				0.34-1.1	1.1-3.7	2.7-108	
Decarbamoylneosaxitoxin (dcNEO)				2.9-4.6	9.6-15	3.3-130	
Deoxydecarbamoylsaxitoxin (doSTX)				0.7-1.2	2.3-3.9	1.5-59	
Saxitoxin (STX)				0.31-0.61	1.0-2.0	3.3-133	
Neosaxitoxin (NEO)				2.4-5.7	8.1-19.0	3.3-131	
Tetrodotoxin (TTX)	Mussels tissues	SLE and IAC	UPLC–MS/MS	0.1 ng/g	0.3 ng/g	0.3-20 ng/mL	[130]

Decarbamoylsaxitoxin (dcSTX)	Oysters, Greensheell mussels, dinoflagellate cultures	SLE and SPE or SLE	LC(HILIC)–MS/MS	33 nmol/kg	110 nmol/kg	-	[131]
Saxitoxin (STX)				18	60	-	
Neosaxitoxin (NEO)				18	59	-	
Decarbamoylgonyautoxin-2 (dcGTX2)				12	40	-	
Decarbamoylgonyautoxin-3 (dcGTX3)				58	190	-	
Gonyautoxin-2 (GTX2)				17	57	-	
Gonyautoxin-3 (GTX3)				5	17	-	
Gonyautoxin-1 (GTX1)				20	68	-	
Gonyautoxin-4 (GTX4)				8	27	-	
Gonyautoxin-4 (GTX5)				23	33	-	
Gonyautoxin-5 (GTX6)				15	13	-	
N-sulfocarbamoyl-C2 (C2)				10	510	-	
N-sulfocarbamoyl-C1 (C1)				3.8	77	-	
Decarbamoylneosaxitoxin (dcNEO)				150	49	-	
Saxitoxin (STX)	Mussels, clam and scallops tissues	SLE and SPE	HILIC-MS/MS	0.33-1.65 μg/kg	1.32-4.96 μg/kg	9.92-158.75 ng/mL	[132]
Neosaxitoxin (NEO)				0.69-2.59	2.07-5.17	10.34-165.47	
Gonyautoxin-1 (GTX1)				4.14-5.52	8.28	12.42-198.74	
Gonyautoxin-2 (GTX2)				2.01-2.82	9.03-11.29	22.57-361.15	
Gonyautoxin-3 (GTX3)				2.55-4.79	4.79-9.58	9.58-153.25	
Gonyautoxin-4 (GTX4)				2.70-3.04	4.05-6.08	4.04-64.82	
N-sulfocarbamoyl-C1 (C1)				2.27-3.33	9.08-9.98	26.96-431.28	
N-sulfocarbamoyl-C2 (C2)				1.35-2.69	2.02-4.03	8.06-128.93	

Pacific Ciguatoxin (P-CTX1B)	Sea urchin, trochus shell, parrotfish, grouper fish, aliquot of culture of Gambierdiscus polynesiensis	SLE, defatting and two consecutive SPE	LC-MS/MS and LC-HRMS	0.0060 μg/kg	0.0125 μg/kg	0.25-2 ng/mL	[133]
Pacific Ciguatoxin (P-CTX3C)				0.050	0.15	12-200	
Anatoxin-a (ANA)	naive channel catfish	SLE and SPE	HILIC–MS/MS	0.004 ng/mL	0.01 ng/mL	0.1-20 ng/mL	[134]
Cylindrospermopsin (CLD)				0.07	0.23	0.5-20	
Saxitoxin (STX)				0.01	0.04	0.1-20	
Microcystin-LA (MLA)			LC-MS/MS	0.02 ng/mL	0.07 ng/mL	0.5-100 ng/mL	
Microcystin-LR (MLR)				0.04	0.13	0.1-100	
Microcystin-LY(MLY)				0.04	0.14	0.5-100	
Microcystin-RR (MRR)				0.06	0.22	0.1-100	
Microcystin-YR (MYR)				0.08	0.28	0.5-100	
Nodularin (NOD)				0.05	0.18	0.5-100	
Domoic acid (DA)	Mussels tissues	SLE and IAC	UHPLC–MS/MS	20 μg/kg	50 μg/kg	50-40000 μg/kg	[125]
Decarbamoylsaxitoxin (dcSTX)	Mussels, Oysters, Cockles	SLE and SPE	UPLC(HILIC)-MS/MS	2.62 μg/kg	20.99 μg/kg	21.0-671.5 μg/kg	[135]
Dicarbamoyl neosaxitoxin (dcNEO)				2.62	5.25	5.25-167.9	
Saxitoxin (STX)				3.10	24.8	24.8-793.7	
Neosaxitoxin (NEO)				6.41	25.66	25.7-821.2	
4-epiTetrodotoxins (4-epiTTX)				7.84	15.68	31.4-1003.3	
11-deoxyTetrodotoxin (11-deoxyTTX)				0.95	1.89	0.9-30.3	
4,9-anhydroTetrodotoxin (4,9-anhydroTTX)				3.73	7.45	3.4-119.2	
Decarbamoylgonyautoxin-2 (dcGTX2)				4.26	8.52	20.3-649.0	
Decarbamoylgonyautoxin-3 (dcGTX3)				0.94	3.75	3.8-120.1	
Gonyautoxin-1 (GTX1)				14.71	29.41	29.4-942.2	
Gonyautoxin-2 (GTX2)				2.54	20.28	20.2-649.0	

Gonyautoxin-3 (GTX3)	6.45	12.9	12.9-412.7
Gonyautoxin-4 (GTX4)	6.48	12.96	6.5-207.3
Gonyautoxin-5 (GTX5)	0.35	2.77	2.8-88.8
Gonyautoxin-6 (GTX6)	0.31	0.62	0.6-19.8
N-sulfocarbamoyl-C1 (C1)	0.08	0.67	0.67-21.6
N-sulfocarbamoyl-C2 (C2)	0.50	2.01	2.0-64.5

Table S4. Selected papers on the analysis of glycoalkaloids in food by liquid chromatography mass spectrometry.

**Toxin**	**Matrix**	**Sample preparation**	**Analytical method**	**LOD**	**LOQ**	**Linearity range**	**Reference**
α-Solanine<br>α-Chaconine<br>Solasodine<br>Demissidine	Potato	SLE and SPE	LC-ESI-MS	10 μg/kg<br>10<br>0.1<br>0.75	25 μg/kg<br>25<br>0.25<br>0.1	25-1000 μg/kg<br>25-1000<br>2.5-100<br>2.5-100	[140]
α-Solanine<br>α-Chaconine	Potato crisps	QuEChERS	UPLC-MS/MS	-<br>-	16 μg/kg<br>31	10-100 ng/mL	[141]
α-Solanine<br>α-Chaconine	Potato	SLE	UHPLC-MS/MS	0.003 μg/g	0.01 μg/g	2-400 μg/L	[142]
Solanidenetriol chacotriose, solanidenediol chacotriose, dehydrosolamargine, solanandaine isomer I, solanandaine, solasonine, robenoside B, malonyl-solanidenediol chacotriose, solamargine isomer, solamargine, solanidatetraenol chacotriose, malonyl-solanandaine malonyl-solanidatetraenol chacotriose, arudonine, malonyl-solamargine isomer, malonyl-solamargine, solanandaine isomer II, solanandaine Isomer III, robenoside B isomer	Eggplant	SLE	LC-FTICR-MS				[145]
Solamargine<br>Solasodine, hydroxysolasodine, hydroxymethylsolasodine, dihydroxysolasodine, dihydroxymethylsolasodine	Solanum scabrum and S. nigrum berries	SLE	UHPLC-MS/MS	3.1 ng/mL	10.2 ng/mL	10.2-5200 ng/mL	[146]
α-Solanine<br>α-Chaconine<br>Solanidine	Potato proteins	SLE and SPE	LC-MS	0.012 μg/mL<br>0.011<br>0.003	0.03 μg/mL<br>0.03<br>0.01	0.03-3 μg/mL<br>0.03-3<br>0.03-1	[147]
α-Solanine<br>α-Chaconine	Potato tissues	SLE and EME	LC-MS/MS	1.5 ng/mL<br>1.2	5.2 ng/mL<br>4.1	5-1000 ng/mL	[144]

Table S5. Selected papers on the analysis of furocoumarins in food by liquid chromatography mass spectrometry.

Toxin	Matrix	Sample preparation	Analytical method	LOD	LOQ	Linearity range	Reference
Xanthotoxol				0.11 mg/kg	0.38 mg/kg		
Heraclenol				0.16	0.55		
Psoralen				0.06	0.18		
Bergaptol				0.26	0.88		
Xanthotoxin				0.05	0.17		
Oxypeucedanin hydrate				0.07	0.23		
Byakangelicin				1.96	6.54		
Isopimpinellin				0.03	0.11		
Heraclenil				0.08	0.26		
Bergapten				0.07	0.23		
Byakangelicol	Citrus peel	SLE	UPLC-MS	0.07	0.23	1-30 µmol/L	[150]
Oxypeucedanin				0.05	0.16		
6',7'-dihydroxybergamottin				0.45	1.51		
Imperatorin				0.4	1.33		
Phellopterin				0.18	0.59		
Cnidilin				0.06	0.19		
Epoxybergamottin				0.71	2.38		
Isoimperatorin				0.19	0.63		
Cnidicin				0.17	0.55		
8-geranyloxypsoralen				0.23	0.78		
Bergamottin				0.03	0.11		
Columbianetin				0.5 ng/mL	1.7 ng/mL	5-1000 ng/mL	
Psoralen				0.33	1	10-5000	
Bergapten				0.3	1	1.1-1100	
Xanthotoxin	Radix Angelicae Pubescentis and related preparations	USLE	LC-MS/MS	0.5	1.5	10.7-1070	[151]
Columbianetin acetate				0.3	1	13-1300	
Imperatorin				0.02	0.2	5-1000	
Osthole				0.01	0.1	12.7-1270	
Isoimperatorin				0.01	0.1	10.3-1030	

Columbianadin				0.17	0.5	13-1300	
Bergaptol	Ruby red grapefruit and juice	QuEChERS	UPLC-MS/MS	5.91 ng/mL	19.69 ng/mL		[152]
Psoralen				0.36	1.21		
8-methoxypsoralen				0.76	2.55		
Bergapten				0.76	2.54		
6',7'-dihydroxybergamottin				1.42	4.74		
Epoxybergamottin				0.45	1.5		
Bergamottin				0.13	0.44		
Bergaptol	Citrus fruit and juices, figs, vegetables, herbs and spices	QuEChERS	UPLC-MS/MS	5.81 µg/kg	19.69 µg/kg	25-10000 ng/mL	[153]
Psoralen				0.36	1.21		
8-methoxypsoralen				0.76	2.55		
Bergapten				0.76	2.54		
6',7'-dihydroxybergamottin				1.42	4.74		
Epoxybergamottin				0.45	1.5		
Bergamottin				0.13	0.44		
Isoimperatorin	Notopterygii Rhizoma et Radix	USLE	UHPLC-MS/MS	0.17 ng/mL	0.5	1-40000 ng/mL	[154]
Psoralen				0.32	0.95	1-400	
Bergapten				0.29	0.9	1-200	
Isoimpinellin				0.15	0.45	1-200	
Bergaptol				0.24	0.75	1-4000	
Cnidilin				5.45	16.5	20-2000	
Pabulelol				0.34	1.04	2-800	
Oxypeucedanin hydrate				0.38	1.15	2-2000	
Demethylfuropinarine				0.28	0.85	1-400	
Notopterol				1.12	3.36	4-40000	
5-dehydronotopterol				0.19	0.57	1-800	
Notoptol				13.33	40	40-8000	
Bergamottin				0.16	0.48	1-20000	
Anhydronotopoloxide				0.55	1.66	2-800	
Nodakenin				0.23	0.7	1-8000	
Angelicin	Bergamot alcoholic beverages, lemon and bergamot juices, earl grey tea, citrus infusion, lemon	SLE or LLE	HPLC-MS/MS	0.0025 mg/L	0.0082 mg/L	0.005-1 mg/L	[155]
Bergamottin				0.0019	0.0066	0.001-5	
Bergapten				0.0015	0.0050	0.001-5	

Byakangelicin	marmalade, home-made *limoncello*	0.0011	0.0038	0.005-5
Byakangelicol		0.002	0.0067	0.005-5
Cnidicin		0.0019	0.0062	0.005-5
Cnidilin		0.0011	0.0036	0.001-5
Epoxybergamottin		0.0014	0.0048	0.005-5
Heraclenin		0.0037	0.0124	0.01-5
Imperatorin		0.0011	0.0036	0.005-5
Imperatorin		0.0013	0.0042	0.001-5
Isobergapten		0.0012	0.0042	0.001-5
Isoimperatorin		0.0003	0.0011	0.001-1
Isopimpinellin		0.0038	0.013	0.005-5
Oxypeucedanin		0.0015	0.0049	0.001-5
Oxypeucedanin hydrate		0.0012	0.0385	0.001-5
Phellopterin		0.0016	0.0050	0.005-5
Psoralen		0.0003	0.0009	0.005-5
Trioxsalen		0.0022	0.0075	0.05-5
8-geranyloxypsoralen		0.0105	0.0352	0.001-5
8-methoxypsoralen		0.0025	0.0082	0.005-1

Table S6. Selected papers on the analysis of cyanogenic glycosides in food by liquid chromatography mass spectrometry.

Toxin	Matrix	Sample preparation	Analytical method	LOD	LOQ	Linearity range	Reference
Amygdalin	Bitter almonds	SLE and dilution	LC-ITMS	200 µg/g	--	--	[170]
Amygdalin	Non-bitter, semi-bitter, and bitter almonds	SLE and SPE	UHPLC-MS/MS	40 µg/kg	130 µg/kg	2.2-200 ng/mL	[171]
Linustatin<br>Neolinustatin	Defatted flaxseeds	alkalinisation and LLE	UHPLC-MS	--	7.621 ng/mL	--	[173]
Amygdalin (and amygdalin isomers)	Bitter almonds (raw, stir-fried and scalded)	SLE and dilution	HPLC-MS/MS	2 µg/mL (amygdalin)	--	0.01–1.03 µg/mL	[172]
Linamarin	Tubers and leaves of Cassava	SLE	LC-MS	0.5 µg/kg	5 µg/kg	5-120 µg/kg	[175]
Linustatin<br>Neolinustatin	Flaxseeds	SLE and hydrolysis	UPLC-HRMS	2000 µg/kg	6600 µg/kg	0.0025-0.5 µg/mL	[174]
Amygdalin<br>Prunasin	Powdered loquat seeds	Defatting, double SLE and dilution	UPLC-MS/MS	22 µg/kg<br>0.015	72 µg/kg<br>0.048	1-100 ng/mL	[176]
Linamarin<br>Linustatin<br>Lotaustralin<br>Neolinustatin<br>Taxiphyllin<br>Dhurrin<br>Amygdalin<br>Prunasin	Fresh cassava roots, fresh bamboo shoots, linseeds, apricot kernels, sorghum rice, almonds, lima beans	SLE and SPE	UHPLC-MS/MS	5 µg/kg<br>5<br>1<br>5<br>5<br>2.5<br>2<br>25	20 µg/kg<br>20<br>5<br>20<br>20<br>10<br>10<br>100	5-200 µg/L<br>5-200<br>5-200<br>5-200<br>5-200<br>5-200<br>5-200<br>25-250	[177]
Amygdalin<br>Dhurrin<br>Prunasin<br>namarin	American elderberry and derived products	SLE and SPE	UHPLC-MS/MS	3 ng/mL<br>3<br>3<br>1	10 ng/mL<br>10<br>5<br>5	10-8000 ng/mL<br>10-6000<br>5-6000<br>5-2000	[178]

Table S7. Selected papers on the analysis of multiclass of natural toxins in food by liquid chromatography mass spectrometry.

Natural toxins classes	Toxin	Matrix	Sample preparation	Analytical method	LOD	LOQ	Linearity range	Other classes	Reference
Mycotoxins	15-Ac-deoxynivalenol, 3-Ac-deoxynivalenol, aflatoxins B1, B2, G1 and G2, altenuene, alternariol, altertoxin I, citrinin, cyclopiazonic acid, deoxynivalenol, deoxynivalenol-3-O-glucoside, diacetoxyscipernol, enniatins A, A1, B and B1, ergocornine, ergocristine, ergocryptine, ergometrine, ergosine, ergotamine, fusarenon X, T-2 and HT-2 toxins, methyl alternarion, monoliformin, neosolaniol,	Almond, banana, bread, coca-cola, coffee, coffee whitener, baby foods, ketchup, meat, milk, olive oil, orange, pizza, red wine, salami sausage, sandwich caviar, sausage, soured milk	SLE or centrifugation for milk samples	UPLC-MS/MS	0.3-160 µg/kg	-	-	Drugs, laxatives, narcotic compounds, pesticides, plant toxins, mushrooms toxins, toxic industry chemicals	[39]

	ochratoxin A, penicillic acid, penitrem A, sterigmatocystin, zearalenone								
Cyanogenic glycoside	Amygdaline				104 µg/kg	-	-		
Marine toxins	domoic acid, microcystin LR, YR and RR, okadaic acid, saxitoxin I				6.5-28 µg/kg	-	-		
Glycoalkaloids	Chaconine, hyoscyamine, solanine, solasonine				0.4 µg/kg	-	-		
Cyanogenic glycoside	Linustatin, neolinustatin	Defatted flaxseeds	alkalinization and LLE	UHPLC-MS	-	7.621 ng/mL	-	Lignan bioactive constituents	[173]
Cyanogenic glycoside	Amygdalin	Medicinal Herbal Extracts of Preventing Hair Loss	SLE and defatting	UPLC-HRMS	0.68 ng/mL	2.26 ng/mLg	50-1000 ng/mL	Bioactive compunds	[179]
Mycotoxins	Ergometrine, ergometrinine, ergosine, ergotamine, ergocornine, α-ergocryptine, β-ergocryptine, ergocristine, ergosinine, ergotaminine,	Cereal based products for infants and young children (breakfast cereals, biscuits and	SLE and cleanup (ultrafiltration)	UPLC-MS/MS	0.1-0.5 µg/kg	--	0.1-50 µg/kg	-	[181]

	ergocorninine, α-ergocryptinine, ergocristinine, chanoclavine-1, ergine, elymoclavine, lysergol, erginine, festuclavine, agroclavine	cookies)							
Tropane alkaloids	Atropine, scopolamine, anisodamine, homatropine, anisodine, aposcopolamine				0.2-0.4 µg/kg	--	0.2-50 µg/kg		
Mycotoxins<br><br>Cyanogenic Glucosides	Mycotoxins<br><br>Linamarin, lotaustralin	Cassava	SLE and dilution	LC-MS/MS	0.002-20.0 µg/kg<br>0.1-2.5 µg/kg	0.006-60.0 µg/kg<br>0.2-8.0 µg/kg	--	-	[184]
Mycotoxins	Aflatoxins B1 and B2, fumonisin B1, ochratoxin A	Apples, apricots, lettuce, onion, cereal-based baby food, wheat, legumes, milk, meat	SLE	HILIC-MS/MS	0.3-3.5 µg/kg	0.8-3.8 µg/kg	2-100 µg/L	Pesticides, plant hormones, veterinary drugs	[185]
Mycotoxins	Beauvericin, enniatins A and B, enniatins A1	Corn, wheat, pasta, rice	SLE	UHPLC-MS/MS	0.2-0.9 µg/kg	0.3-2.6 µg/kg	1-100 µg/kg (pasta) 2-400 µg/kg (corn,	Bacterial toxin	[180]

	and B1						wheat, rice)		
Tropane alkaloids	Atropine, scopolamine	Oat and wheat	SLE	2D-LC-MS/MS	0.05-0.12 µg/kg	0.16-0.40 µg/kg	--	Growth regulators	
Mycotoxins	Aflatoxins B1, B2, G1 and G2, deoxynivalenol, T2-toxin, HT-2 toxin, ochratoxin A, zearalenone				0.01-7.66 µg/kg	0.04-25.52 µg/kg	0.01-1000µg/kg		
Pyrrolizidine alkaloids	Erucifoline, heliotrine, echimidine, europine, jacobine, retrorsine, lasiocarpine-n-oxide, monocrotaline, seneciphylline, seneciphylline-n-oxide, senkirkine, trichodesmine				0.05-0.20 µg/kg	0.18-0.68 µg/kg	0.2-50 µg/kg		
Cyanogenic glycoside	Linustatin, neolinustatin	Flaxseeds coated with herbal extracts	USLE and hydrolysis	UPLC-HRMS	2 µg/g	6.6 µg/g	0.0025-0.5 µg/mL	Lignan bioactive constituents	[174]
Tropane alkaloids	Atropine, scopolamine	Corn semolina, wheat flour, cereal	SLE, QuECHERS and defatting	LC-MS/MS	-	0.50 µg/kg	--	-	[108]
Mycotoxins	Fumonisins B1 and B2, aflatoxins B1,				-	0.20-100 µg/kg	--		

	B2, G1 and G2, deoxynivalenol, ochratoxin A, zearalenone, T-2 and HT-2 toxins	flakes, oat flour, quinoa flour, soy-beans, infant cereals							
Mycotoxins	Ergometrine, ergometrinine, ergosine, ergotamine, ergocornine, α-ergocryptine, β-ergocryptine, ergocristine, ergosinine, ergotaminine, ergocorninine, α-ergocryptinine, ergocristinine, chanoclavine-1, ergine, elymoclavine, lysergol, erginine, festuclavine, agroclavine	Bread (wheat, multigrain, rye and wheat-rye breads)	SLE and ultrafiltratio n	LC-MS/MS	0.1-0.4 μg/kg	0.3-1.2 μg/kg	-	-	[182]
Tropane alkaloids	Atropine, scopolamine, anisodamine, homatropine, anisodine, aposcopolamine				0.2-0.3 μg/kg	0.5-1.0 μg/kg	-		

Furocoumarins	Psoralen, bergaptol, xanthotoxin, bergapten, 6',7'-dihydroxybergamottin, imperatorin, isoimperatorin, 6',7'-epoxybergamottin	Pummelo fruits	USLE	UHPLC-MS/MS	0.03-0.45 µg/L	0.13-1.50 µg/L	0.19-96.15 µg/L	Coumarins (4), Flavonoids (27), phenolic compounds (8)
Mycotoxins	Aflatoxins B1, B2, G1 and G2, altenuene, alternariol, alternariol monomethyl ether, citrinin, deoxynivalenol, deoxynivalenol-3-glucoside, 15-acetyldeoxynivalenol, 3-acetyldeoxynivalenol, diacetoxyscirpenol, fumonisins B1, B2 and B3, hydrolyzed fumonisins B1 and B2, fusarenon X, T-2 and HT-2 toxins, neosolaniol,	Wheat (for validation) and barley, rice, oat, spelt, rye	Dilute and shoot	2D-LC-MS/MS	-	1-300 µg/kg	1-8000 µg/kg	Growth regulators

	nivalenol, ochratoxin A, sterigmatocystin, tentoxin, zearalenone, zearalenone-14-glucoside, zearalenone-14-sulfate, zearalenone-14,16-disulfate, α-zearalenol, α-zearalenol-14-glucoside, α-zearalenol-14-sulfate, β-zearalenol, β-zearalenol-14-glucoside, β-zearalenol-14-sulfate, zearalanone, zearalanone-14-glucoside, α-zearalanol, β-zearalanol			
Tropane alkaloids	Atropine, scopolamine	-	5 µg/kg	5-200 µg/kg
